# Nucleoporin TPR integrates MAPK signaling with mitogen-induced transcriptional programs

**DOI:** 10.1038/s41419-026-08760-8

**Published:** 2026-04-24

**Authors:** Jin Liu, Yifan Zheng, Yi Xiong, Runhua Ma, Zihao Lin, Haikun Lin, Miguel Andújar-Sánchez, Jirina Bartkova, Jian Liu, Marco Foiani, Jiri Bartek, Martin Kosar

**Affiliations:** 1 Department of Burns, the Second Affiliated Hospital of Zhejiang University School of Medicine, and the Centre for Infection, Immunity, and Cancer (IIC) at Zhejiang University–University of Edinburgh Institute, Zhejiang University School of Medicine, Haining, China; 2 Edinburgh Medical School: Biomedical Sciences, College of Medicine and Veterinary Medicine, The University of Edinburgh, Edinburgh, UK; 3 Pathology Department, Complejo Hospitalario Universitario Insular Materno Infantil, Las Palmas de Gran Canaria, Spain; 4 Division of Genome Biology, Department of Medical Biochemistry and Biophysics, Science for Life Laboratory, Karolinska Institutet, Stockholm, Sweden; 5 Danish Cancer Institute, Danish Cancer Society, Copenhagen, Denmark; 6 Centre for Infection, Immunity, and Cancer (IIC) at Zhejiang University–University of Edinburgh Institute, Zhejiang University School of Medicine, Haining, China; 7 Biomedical and Health Translational Research Center of Zhejiang Province, Haining, China; 8 IFOM, Fondazione Istituto FIRC di Oncologia Molecolare, Milano, Italy

**Keywords:** Cell biology, Cancer

## Abstract

The nuclear pore complex (NPC) component TPR has emerged as a multifunctional scaffold implicated in mitosis, chromatin organization, mRNA export, and genome stability. However, TPR’s role in mitogenic signal transduction remains largely unexplored. Here, we investigate whether nucleoporin TPR functions as a MAPK-regulated nuclear component that modulates mitogenic signals initiated at the plasma membrane—including EGFR activation—and their transcriptional output. Transcriptomic profiling reveals that TPR depletion reshapes EGF-induced, MAPK-responsive gene expression, including altered expression of MAPK pathway components and enhanced induction of the immediate-early gene *FOS*. Functionally, TPR-depleted cells exhibit increased *FOS* induction upon EGF stimulation and altered EGF-driven cell-cycle progression. Using a novel phospho-specific monoclonal antibody, we show that TPR is phosphorylated at Ser2155 following EGFR activation via the canonical RAS–RAF–MEK–ERK MAPK cascade, placing TPR downstream of MAPK pathway activation. This phosphorylation is suppressed by clinically used EGFR and BRAF inhibitors and, conversely, is constitutively induced by oncogenic RAS and BRAF, indicating that Ser2155 phosphorylation reflects MAPK pathway activity. In vivo, CRISPR/Cas9-engineered *Tpr* haploinsufficient mice show changes in MAPK pathway regulatory gene expression in bulk spleen RNA-seq, consistent with findings in human cells, and enhanced *Fos* induction in splenocytes upon CD3/CD28 stimulation, together suggesting a conserved association between TPR levels and altered MAPK-related transcriptomic profiles. Finally, immunohistochemical analysis reveals elevated TPR phosphorylation in serous ovarian carcinoma and heterogeneous phosphorylation patterns in triple-negative breast cancer, two tumor types frequently characterized by MAPK pathway hyperactivation. Together, these findings uncover a previously unappreciated role for TPR as a MAPK-responsive nuclear factor and support a model in which NPC-associated components fine-tune mitogen-induced transcriptional responses.

## Introduction

Nuclear pore complexes (NPCs) are essential macromolecular structures embedded in the nuclear envelope, functioning as selective channels for nucleocytoplasmic transport. Comprising approximately 30 distinct nucleoporins (NUPs), NPCs not only mediate nucleocytoplasmic transport but also perform transport-independent functions, including the regulation of chromatin organization, gene expression, and mitotic progression [1–3]. Aberrant expression or structural alterations of nucleoporins have been implicated in a wide range of human diseases, including autoimmune disorders, cardiovascular and neurological conditions, and cancer [4].

Human nucleoporin TPR (Translocated promoter region protein) is a large scaffold protein characterized by a ~1600-amino-acid coiled-coil N-terminal domain and a ~800-amino-acid C-terminal domain containing a nuclear localization signal [5]. The N-terminal domain anchors TPR to the nuclear basket of the nuclear pore complex (NPC) by interacting with nucleoporin Nup153, positioning TPR on the nucleoplasmic side of the NPC [6]. TPR regulates several essential cellular processes, including the mitotic spindle checkpoint [7], chromatin organization relevant to HIV replication [8], mRNA export [9], and protection from RNA-mediated replication stress [10].

Previously published analyses have reported aberrant *TPR* mRNA and TPR protein levels across multiple tumor types, including ovarian and breast cancers, indicating that TPR dysregulation may be associated with oncogenic pathways [10, 11]. Somatic mutations in TPR, including recurrent substitution at Ser2155, have also been identified in endometrial, colorectal, and skin cancers [11]. Together, these observations suggest that altered TPR expression or regulation may be associated with oncogenic signaling programs, yet the mechanistic contribution of TPR to cancer-relevant signaling pathways remains unclear.

Growing evidence points to a potential link between TPR and mitogenic signaling. The epidermal growth factor receptor (EGFR)–RAS–RAF–MEK–ERK MAPK cascade is an important regulator of cell proliferation, differentiation, survival, and mitogen-responsive transcription, and its aberrant activation is a hallmark of many cancers [12–14]. Notably, mass spectrometry-based phosphoproteomic screens have identified Ser2155 of TPR as rapidly phosphorylated following epidermal growth factor (EGF) stimulation [15, 16]. TPR has also been proposed as a putative substrate of ERK1/2 kinases, and co-immunoprecipitation experiments indicate physical interaction between TPR and ERK1/2 [17, 18]. These findings raise the possibility that TPR is regulated downstream of MAPK signaling, although whether and how TPR contributes to MAPK-dependent transcriptional responses has remained unresolved.

Beyond EGFR-driven signaling, the RAS–RAF–MEK–ERK MAPK cascade is also activated in multiple other physiological contexts including in immune B and T cells through their antigen receptor engagement [19, 20], providing EGFR-independent scenarios to examine MAPK-driven transcriptional responses.

In this study, we investigate whether TPR acts as a MAPK-regulated nuclear component that modulates transcriptional responses to mitogenic stimulation. We combine transcriptomic analyses, biochemical characterization of TPR regulation, and genetic perturbation in both cultured cells and mouse models to define how TPR participates in MAPK-associated gene expression programs. To assess clinical relevance, we immunohistochemically examine TPR phosphorylation in human carcinoma types commonly linked to MAPK pathway activation.

Together, our results indicate that the nucleoporin TPR is not solely a structural component of the nuclear pore complex but also contributes to nuclear transcriptional responses downstream of MAPK pathway activation, linking mitogenic inputs—including EGFR and TCR/CD3 stimulation—to changes in gene expression. These findings establish TPR as a MAPK-responsive nuclear factor and provide a framework for understanding how NPC components fine-tune signal-dependent transcription in physiological and disease contexts.

## Results

### Distinct transcriptional responses to EGF stimulation and TPR depletion in HeLa cells

To investigate whether TPR modulates transcriptional responses downstream of EGFR activation, we performed RNA sequencing (RNA-seq) in HeLa cells following TPR depletion (siTPR) or transfection with a control siRNA (siCTRL) under mock-treated and EGF-stimulated conditions.

**Fig. 1:**
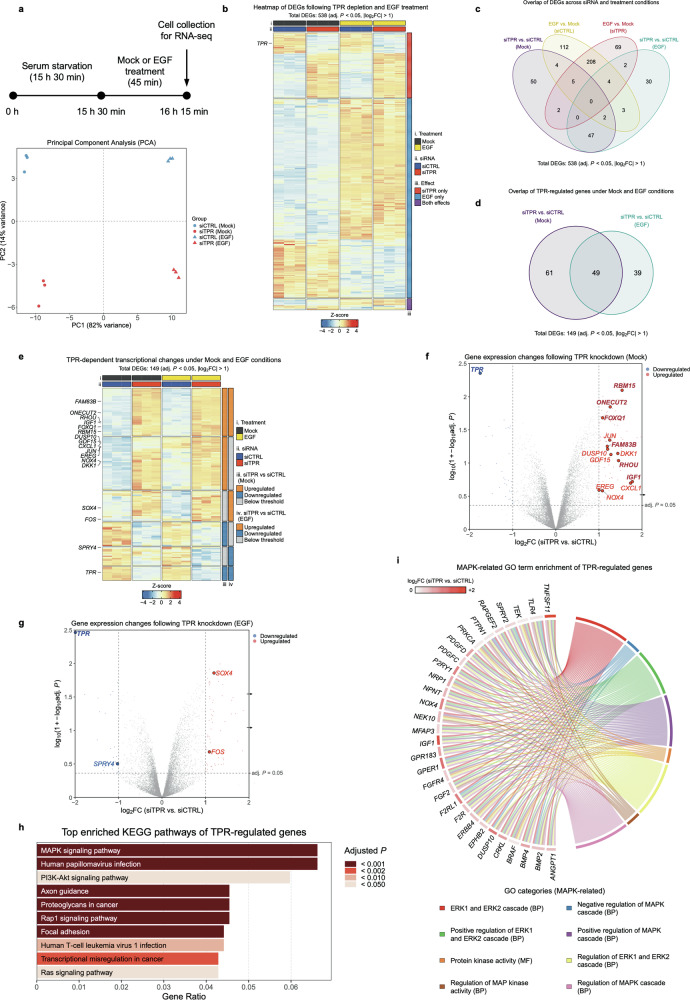
TPR depletion alters EGF-induced transcriptional programs in HeLa cells. **a** Upper panel: Schematic overview of the RNA-seq workflow. HeLa cells were transfected with control siRNA (siCTRL) or TPR siRNA (siTPR), serum-starved for 15 h 30 min, and then mock-treated or stimulated with EGF for 45 min before RNA extraction for RNA-seq analysis. Bottom panel: Principal component analysis (PCA) of transcriptomic profiles from RNA-seq data. PC1 (82% variance) separates samples by EGF stimulation, while PC2 (14% variance) distinguishes between siCTRL and siTPR samples, indicating that TPR depletion modulates both mock-treated and EGF-stimulated transcriptional programs. **b** Heatmap showing 538 differentially expressed genes (DEGs) across all experimental conditions (adj. *P* < 0.05 and |log_2_FC| > 1). Samples include siCTRL and siTPR HeLa cells treated with either mock or EGF. Hierarchical clustering reveals distinct transcriptomic signatures associated with TPR depletion alone, EGF stimulation alone, or their combined effects. Annotation color bars indicate treatment (mock or EGF), siRNA condition (siCTRL or siTPR), and effect category (siTPR only, EGF only, or both effects). adj. *P*, adjusted *P* value; log_2_FC, log_2_ fold change. **c** Venn diagram illustrating the overlap of DEGs (adj. *P* < 0.05 and |log_2_FC| > 1) from four pairwise comparisons: (1) EGF vs. mock (siCTRL), (2) EGF vs. mock (siTPR), (3) siTPR vs. siCTRL (under mock-treated conditions), and (4) siTPR vs. siCTRL (under EGF-stimulated conditions). **d** Venn diagram showing the overlap of DEGs (adj. *P* < 0.05 and |log_2_FC| > 1) upon TPR depletion under mock and EGF-stimulated conditions (siTPR vs. siCTRL comparisons). Of 149 total DEGs, 61 were unique to mock conditions, 49 were shared between both conditions, and 39 were unique to EGF-stimulated conditions. **e** Heatmap showing 149 DEGs (adj. *P* < 0.05 and |log_2_FC| > 1) identified upon TPR depletion under mock- and EGF-stimulated conditions. The heatmap highlights transcriptional changes associated with loss of TPR under both mock-treated and EGF-stimulated states. The right-side color bar indicates expression changes under mock or EGF conditions: upregulated (adj. *P* < 0.05 and log₂FC> 1), downregulated (adj. *P* < 0.05 and log₂FC < −1), and below threshold (adj. *P* > 0.05 or |log₂FC| < 1). **f** Volcano plot showing differential gene expression following TPR depletion in HeLa cells under mock-treated conditions (siTPR vs. siCTRL). Significantly upregulated genes are shown in red, and significantly downregulated genes are shown in blue (adj. *P* < 0.05 and |log_2_FC| > 1). Selected MAPK-associated genes are annotated. Dot color indicates the direction of regulation (red, up; blue, down). Gene label color denotes condition specificity: labels matching the dot color indicate genes regulated exclusively under mock-treated conditions, whereas darker red or darker blue labels in bold indicate genes regulated under both mock-treated and EGF-stimulated conditions. The x-axis represents log₂ fold change, while the y-axis shows a double-logarithmic transformation of adjusted *P* values [log₁₀(1 + −log₁₀(adj. *P*))]. The arrow indicates a gene with a value exceeding the plotted axis range. **g** Volcano plot showing differential gene expression following TPR depletion in HeLa cells under EGF-stimulated conditions (siTPR vs. siCTRL). Significantly upregulated genes are shown in red, and significantly downregulated genes are shown in blue (adj. *P* < 0.05 and |log₂FC| > 1). Selected MAPK-associated genes are annotated. Dot color indicates the direction of regulation (red, up; blue, down). Gene label color denotes condition specificity: labels matching the dot color indicate genes regulated exclusively under EGF-stimulated conditions, whereas the darker blue label in bold indicates the gene regulated under both mock-treated and EGF-stimulated conditions. The x-axis represents log₂ fold change, while the y-axis shows a double-logarithmic transformation of adjusted *P* values [log₁₀(1 + −log₁₀(adj. *P*))]. Arrows indicate genes with values exceeding the plotted axis range. **h** KEGG pathway enrichment analysis of TPR-regulated genes under mock-treated conditions. Bars represent the top enriched pathways ranked by gene ratio, and bar color indicates the adjusted *P* value. **i** Chord diagram showing significantly enriched MAPK-related GO terms (adj. *P* < 0.05) following TPR depletion under mock-treated conditions, as determined by gene set enrichment analysis (GSEA). Circular nodes represent GO terms and individual genes, with connecting ribbons indicating gene–term associations. BP biological process; MF molecular function.

Cells were serum-starved for 15 h 30 min and then stimulated with EGF for 45 min prior to RNA extraction (Fig. 1a, *upper panel*). A total of three independent biological replicates were analyzed.

Principal component analysis (PCA) revealed clear segregation of samples by condition. PC1 (82% variance) separated EGF-stimulated from unstimulated samples, while PC2 (14% variance) separated siTPR from siCTRL samples, indicating that both EGF stimulation and TPR depletion independently elicit robust and distinct transcriptomic changes (Fig. 1a, *bottom panel*).

Differential expression analysis (adjusted *P* value (adj. *P*) < 0.05 and |log_2_ fold change (log_2_FC)| > 1) identified 538 differentially expressed genes (DEGs) across all conditions (Fig. 1b). Hierarchical clustering revealed transcriptional signatures associated with TPR depletion, EGF stimulation, and their combined effects. A Venn diagram further defined shared and unique DEG sets (Fig. 1c), illustrating the differences in gene expression between siTPR and control cells under both mock-treated and EGF-stimulated conditions.

Among the 538 DEGs identified across all conditions, 149 genes were upregulated or downregulated upon TPR depletion. Based on their response patterns across conditions, these genes were classified into three categories by overlap analysis (Fig. 1d): 61 genes differentially expressed only under mock-treated conditions, 49 genes differentially expressed under both mock- and EGF-stimulated conditions, and 39 genes differentially expressed only following EGF stimulation.

A heatmap of these 149 TPR-regulated DEGs displayed clear clustering by siRNA treatment and EGF stimulation state (Fig. 1e).

To visualize transcriptional changes induced by TPR depletion, volcano plots were generated separately for mock-treated and EGF-stimulated cells by comparing siTPR and siCTRL samples within each condition. The mock-condition volcano plot (Fig. 1f) highlights all genes significantly regulated by TPR depletion under mock-treated conditions in color, including 61 mock-specific genes and 49 shared genes that are regulated by TPR depletion under both mock- and EGF-stimulated conditions. *TPR* and selected MAPK-associated genes are additionally labeled.

By contrast, the EGF-condition volcano plot (Fig. 1g) highlights all genes significantly regulated by TPR depletion under EGF-stimulated conditions in color, including genes regulated by TPR depletion under both mock- and EGF-stimulated conditions (49 shared genes), as well as genes that are regulated specifically upon EGF stimulation (39 EGF-specific genes). As in Fig. 1f, *TPR* and selected MAPK-associated genes are additionally labeled.

To identify pathways influenced by TPR loss, we performed Kyoto Encyclopedia of Genes and Genomes (KEGG) enrichment analysis, which revealed enrichment of MAPK and PI3K-Akt signaling—key pathways activated by mitogenic stimulation—along with additional categories such as “Transcriptional misregulation in cancer” (Fig. 1h).

Gene Ontology (GO) analysis further identified multiple significantly enriched GO terms associated with MAPK signaling (adj. *P* < 0.05), with a chord diagram illustrating the contribution of individual genes to MAPK-related GO categories (Fig. 1i).

Together, these data define three groups of genes whose expression differs between TPR-depleted and control cells under mock-treated and EGF-stimulated conditions, thereby providing the basis for subsequent analyses.

### Upregulation of MAPK-associated genes upon TPR depletion in mock-treated HeLa cells

Among the 61 genes differentially expressed in TPR-depleted HeLa cells under mock-treated conditions (Fig. 1d, *left region*), many are linked to MAPK-associated signaling. The volcano plot (Fig. 1f) shows significantly up- or downregulated genes (adj. *P* < 0.05 and |log_2_FC| > 1) in color, with selected MAPK-associated genes annotated. *TPR* itself is among the most strongly downregulated transcripts, providing internal validation of efficient TPR knockdown.

Notably, *JUN*, encoding the AP-1 transcription factor c-Jun, was upregulated. As a core MAPK-responsive transcription factor, c-Jun contributes to growth factor-induced transcription [12]. We also observed significantly increased expression of *EREG* (Epiregulin), an EGFR ligand that activates the pathway and contributes to tumor progression [21]. *CXCL1*, a pro-tumorigenic chemokine that can promote EGFR transactivation in several cancer types [22, 23], was likewise significantly upregulated.

We additionally detected increased expression of MAPK regulators, including *DUSP10*, a dual-specificity phosphatase that negatively regulates ERK activity [24]. Other significantly upregulated genes included *NOX4*, an NADPH oxidase involved in ROS production and the enhancement of EGFR signaling [25]; *GDF15*, which can activate MAPK signaling through ERBB2 receptor [26]; and *DKK1*, a gene transcriptionally regulated downstream of EGFR pathway activation [27].

Collectively, these results identify multiple MAPK-associated genes whose basal expression differs between TPR-depleted and control cells under unstimulated conditions.

### Upregulation of MAPK-associated genes upon TPR depletion under both mock-treated and EGF-stimulated conditions

In addition to the genes altered only in mock-treated cells, we identified 49 genes that were differentially expressed upon TPR depletion under both mock-treated and EGF-stimulated conditions, indicating a shared transcriptional response to TPR loss across conditions (Fig. 1d, *overlap region*). The volcano plot (Fig. 1f) shows significantly upregulated or downregulated genes (adj. *P* < 0.05 and |log_2_FC| > 1) in color, with selected MAPK-associated genes annotated.

*FOXQ1*, a transcription factor that binds the EGFR promoter and regulates its transcription [28], was significantly upregulated. We also observed significantly increased expression of *RHOU*, an atypical Rho GTPase that associates with activated EGFR via GRB2 and promotes AP-1 transcriptional activity and cell migration [29]. *ONECUT2*, a transcriptional regulator implicated in RAS-driven lung adenocarcinoma [30], and *FAM83B*, an effector of EGFR- and RAS-mediated transformation [31], were likewise upregulated. TPR depletion also resulted in upregulated *IGF1*, a ligand for the IGF-1R implicated in cross-talk with EGFR and resistance to EGFR-targeted therapies [32]. Furthermore, TPR-depleted cells showed upregulated *RBM15*, known to promote MAPK hyperactivation by enhancing m^6^A modification and stability of *STYK1* mRNA, thereby increasing ERK signaling activity [33].

Together, these results identify multiple MAPK-associated genes whose expression differs between TPR-depleted and control cells under both mock-treated and EGF-stimulated conditions.

### Altered expression of MAPK-associated genes upon TPR depletion in EGF-stimulated cells

In addition to the genes differentially expressed under mock-treated conditions and those shared between mock-treated and EGF-stimulated conditions, we identified 39 genes that were differentially expressed specifically upon EGF stimulation in TPR-depleted HeLa cells (Fig. 1d, *right region*). The volcano plot (Fig. 1g) highlights genes significantly upregulated or downregulated (adj. *P* < 0.05 and |log_2_FC| > 1) upon TPR depletion under EGF-stimulated conditions in color, with selected MAPK-associated genes that are specifically regulated upon EGF stimulation annotated.

Among these genes, *FOS*, a canonical ERK-responsive immediate-early gene, was significantly upregulated in TPR-depleted cells following EGF stimulation. Its protein product, c-Fos, forms the AP-1 transcription complex together with c-Jun [12]. *SOX4*, a transcription factor that regulates EGFR and MAPK family kinases [34, 35], was also upregulated. In contrast, *SPRY4*, a well-established negative regulator of MAPK signaling [36], was significantly downregulated. Given the role of *SPRY4* in constraining MAPK pathway activity [37], these changes indicate that TPR loss affects both positive and negative regulatory nodes within the MAPK pathway.

Collectively, these results identify MAPK-associated genes whose expression differs between TPR-depleted and control cells following EGF stimulation.

### Elevated EGF-induced *FOS* expression upon TPR depletion

**Fig. 2:**
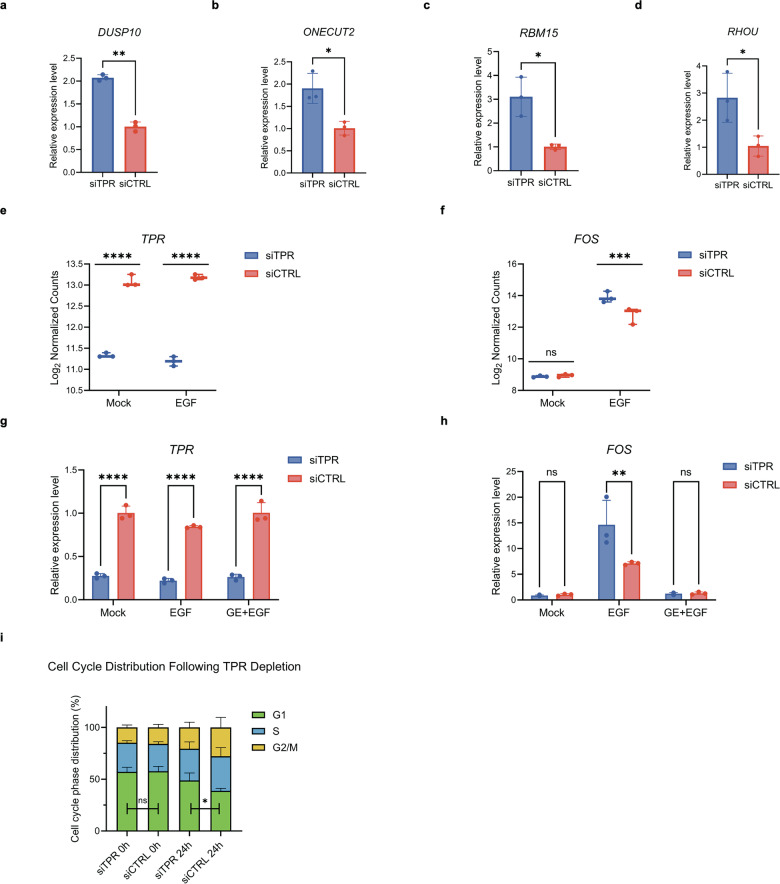
TPR depletion enhances *FOS* expression and alters EGF-induced cell-cycle progression. **a** Quantitative real-time PCR (qPCR) validation of *DUSP10* mRNA expression in siTPR and siCTRL HeLa cells under mock conditions. Data are presented as mean ± SD (*n* = 3 biological replicates per condition). Statistical analysis was performed using a two-tailed paired *t*-test; **P < 0.01. **b** Quantitative real-time PCR (qPCR) validation of *ONECUT2* mRNA expression in siTPR and siCTRL HeLa cells under mock conditions. Data are presented as mean ± SD (*n* = 3 biological replicates per condition). Statistical analysis was performed using a two-tailed paired *t*-test; *P < 0.05. **c** Quantitative real-time PCR (qPCR) validation of *RBM15* mRNA expression in siTPR and siCTRL HeLa cells under mock conditions. Data are presented as mean ± SD (*n* = 3 biological replicates per condition). Statistical analysis was performed using a two-tailed paired *t*-test; *P < 0.05. **d** Quantitative real-time PCR (qPCR) validation of *RHOU* mRNA expression in siTPR and siCTRL HeLa cells under mock conditions. Data are presented as mean ± SD (*n* = 3 biological replicates per condition). Statistical analysis was performed using a two-tailed paired *t*-test; *P < 0.05. **e** Assessment of *TPR* mRNA expression upon TPR depletion under mock and EGF-stimulated conditions by RNA-seq. *TPR* mRNA levels were significantly reduced upon siTPR treatment under both conditions, confirming efficient knockdown. Data are presented as mean ± SD (*n* = 3 biological replicates per condition). **** adjusted *P* value < 0.0001. **f** Assessment of *FOS* mRNA expression upon TPR depletion under mock and EGF-stimulated conditions by RNA-seq. FOS mRNA induction in response to EGF stimulation was significantly enhanced upon TPR depletion, whereas no significant difference was observed between control and TPR-depleted cells under mock conditions. Data are presented as mean ± SD (*n* = 3 biological replicates per condition). ns, not significant; *** adjusted *P* value < 0.001. **g** Quantitative real-time PCR (qPCR) validation of *TPR* mRNA expression in siTPR and siCTRL HeLa cells under mock conditions or after 1 h pretreatment with DMSO (vehicle control) or gefitinib (an EGFR tyrosine kinase inhibitor) followed by EGF stimulation for 45 min. qPCR analysis confirms efficient TPR knockdown under all conditions tested. Data are presented as mean ± SD (*n* = 3 biological replicates per condition). Statistical analysis was performed using two-way ANOVA followed by Šídák’s multiple-comparisons test; ****P < 0.0001. **h** Quantitative real-time PCR (qPCR) validation of *FOS* mRNA expression in siTPR and siCTRL HeLa cells under mock conditions or after 1 h pretreatment with DMSO (vehicle control) or gefitinib (an EGFR tyrosine kinase inhibitor) followed by EGF stimulation for 45 min. *FOS* mRNA expression was significantly increased in siTPR cells following EGF stimulation, and this induction was suppressed by gefitinib treatment, whereas it remained unchanged under mock conditions. Data are presented as mean ± SD (*n* = 3 biological replicates per condition). Statistical analysis was performed using two-way ANOVA followed by Šídák’s multiple-comparisons test; ns, not significant; **P < 0.01. **i** Cell cycle distribution of control and TPR-depleted HeLa cells. HeLa cells transfected with control siRNA (siCTRL) or TPR siRNA (siTPR) were serum-starved and either analyzed at 0 h or stimulated with EGF for 24 h before analysis by flow cytometry. Stacked bar charts show the relative proportions of cells in G1 (green), S (blue), and G2 (yellow) phases under each condition. Data are presented as mean ± SD (*n* = 3 biological replicates per condition). Statistical analysis was performed using a two-tailed paired *t*-test; ns, not significant; *P < 0.05.

To validate the transcriptomic changes identified by RNA-seq, we first examined four selected candidate target genes (*DUSP10*, *ONECUT2*, *RBM15*, and *RHOU*). Independent qPCR experiments confirmed significant upregulation of all four transcripts following TPR depletion (Fig. 2a–d). Consistent with the volcano plot shown in Fig. 1g, RNA-seq-derived expression plots demonstrated the changes in *TPR* and *FOS* under mock- and EGF-stimulated conditions (Fig. 2e, f).

To further validate these findings, HeLa cells were serum-starved for 16 h, pretreated with either DMSO or gefitinib, a clinically approved EGFR tyrosine kinase inhibitor [38], for 1 h, and subsequently stimulated with EGF for 45 min. qPCR confirmed robust depletion of *TPR* under both mock- and EGF-stimulated conditions (Fig. 2g). Consistent with the RNA-seq results, *FOS* mRNA was markedly upregulated in TPR-depleted cells after EGF stimulation (Fig. 2h), and this increase was suppressed by gefitinib, indicating a requirement for EGFR kinase activity.

### Altered EGF-induced cell-cycle progression in TPR-depleted HeLa cells

Based on RNA-seq results showing altered expression of immediate-early genes upon TPR depletion, we examined whether TPR influences EGF-induced cell-cycle progression. TPR-depleted (siTPR) and control (siCTRL) HeLa cells were serum-starved and analyzed at baseline (0 h) and after 24 h of EGF stimulation.

At baseline, TPR-depleted and control cells displayed comparable G1-phase populations (Fig. 2i), indicating that TPR depletion does not affect the cell-cycle profile under growth factor-deprived conditions. Following 24 h of EGF stimulation, TPR-depleted cells exhibited a higher proportion of G1-phase cells compared with control cells (Fig. 2i), indicating that TPR depletion alters the overall cell-cycle response to EGF.

### Generation and validation of a phospho-specific TPR (Ser2155) antibody

**Fig. 3:**
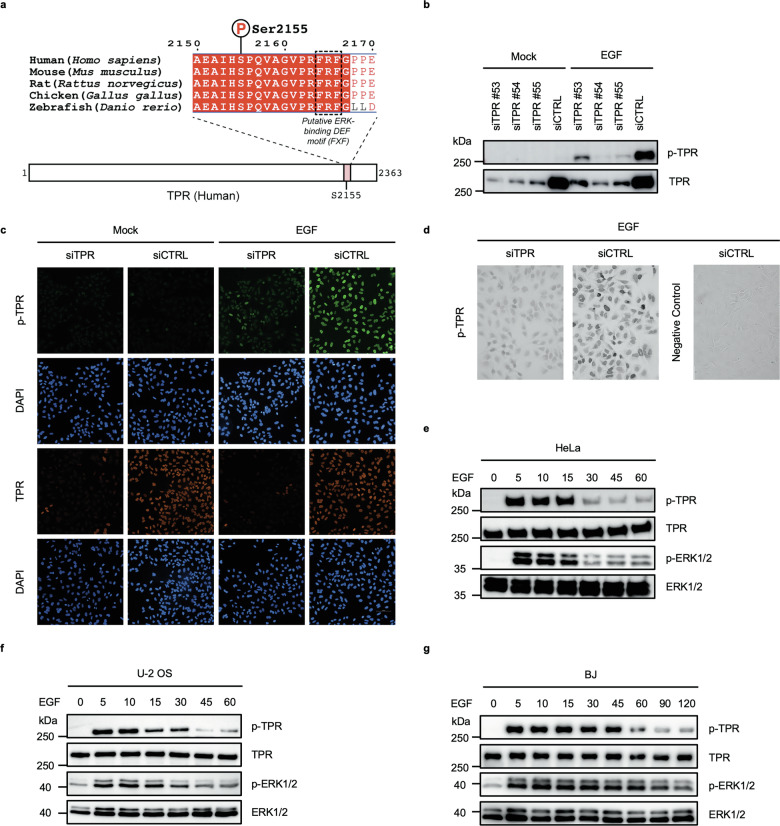
Phosphorylation of TPR at Ser2155 in response to EGF stimulation. **a** Conservation of a putative ERK-regulated site in the C-terminus of TPR. Schematic representation of human TPR (residues 1–2363) indicating the position of Ser2155 near the C-terminus. A multiple sequence alignment of the region surrounding Ser2155 across five vertebrate species (human, mouse, rat, chicken, and zebrafish) highlights strong evolutionary conservation. Ser2155, corresponding to a putative phosphorylation site, is indicated, and the adjacent putative ERK-binding DEF motif (FXF) is shown. **b** Immunoblot analysis of phospho-TPR-Ser2155 (p-TPR) and total TPR in HeLa cells transfected with three independent siRNAs targeting *TPR* (siTPR #53, #54, and #55) or control siRNA (siCTRL). Cells were mock-treated or EGF-stimulated for 5 min. Data are representative of three independent experiments. **c** Immunofluorescence microscopy showing the nuclear localization of phospho-TPR-Ser2155 (p-TPR, green) and total TPR (orange) in siCTRL- and siTPR-transfected HeLa cells following 5 min EGF stimulation or mock treatment. DAPI (blue) was used to visualize nuclei. Data are representative of three independent experiments. Scale bar: 100 µm. **d** Immunohistochemical detection of phospho-TPR-Ser2155 (p-TPR) in paraffin-embedded HeLa cells following 5 min EGF stimulation, comparing siTPR-transfected cells with siCTRL-transfected cells. An antibody specific for phosphorylated TPR at Ser2155 was used to stain p-TPR, while nonimmune serum was used as a negative control. Data are representative of three independent experiments. Scale bar: 50 µm. **e** Time-course analysis of TPR phosphorylation at Ser2155 in HeLa cells stimulated with EGF for 0–60 min. TPR phosphorylation was detected using a phospho-TPR-Ser2155 (p-TPR) antibody. Total TPR served as a loading control, and phosphorylation kinetics were compared with the dynamics of ERK1/2 activation. Data are representative of three independent experiments. **f** Time-course analysis of phospho-TPR-Ser2155 (p-TPR), total TPR, phospho-ERK1/2, and total ERK1/2 in U-2 OS cells treated with EGF for the indicated times (0–60 min). Data are representative of three independent experiments. **g** Time-course analysis of phospho-TPR-Ser2155 (p-TPR), total TPR, phospho-ERK1/2, and total ERK1/2 in non-cancerous human BJ fibroblasts treated with EGF for the indicated times (0–120 min). Data are representative of three independent experiments.

Given that previous mass spectrometry-based phosphoproteomic screens identified Ser2155 of TPR as an EGF-responsive phosphorylation site [15, 16], we examined whether Ser2155 is phosphorylated upon mitogenic stimulation. As shown in Fig. 3a, Ser2155 is located within the C-terminal region of TPR adjacent to a putative ERK-binding DEF motif (FXF), a known ERK-docking element [39], and this region is highly conserved across vertebrates.

Because no phospho-specific antibodies for this site were available, we generated a mouse monoclonal antibody that selectively recognizes TPR phosphorylated at Ser2155. To validate specificity, HeLa cells were transfected with siCTRL or three independent siRNAs targeting *TPR*. After 5 min of EGF stimulation, phospho-TPR (Ser2155) signal was readily detected in control cells but strongly reduced upon TPR depletion, confirming antibody specificity by immunoblotting (Fig. 3b). All full-length uncropped original western blots are provided in the file Original Data.

In immunofluorescence analyses, total TPR localized to the nuclear envelope under both mock- and EGF-treated conditions, whereas phospho-TPR levels increased robustly after 5 min of EGF stimulation and were lost in siTPR cells (Fig. 3c). Confocal imaging confirmed that phospho-TPR signals originate from cells retaining total TPR and are enriched at the nuclear periphery (Supplementary Fig. 1a). The antibody also performed robustly in tissue immunohistochemistry (Supplementary Fig. 1b) and immunoperoxidase-based cytochemistry, where EGF-induced phospho-TPR signal was abolished upon TPR depletion (Fig. 3d). Peptide-blocking assays further demonstrated specificity, as the signal was eliminated only when the phospho-TPR antibody was pre-incubated with the phosphopeptide and not with the non-phosphorylated peptide (Supplementary Fig. 1c–e).

Together, these results validate the phospho-TPR (Ser2155) antibody for detecting endogenous TPR phosphorylation across multiple experimental assays.

### Rapid phosphorylation of TPR at Ser2155 following EGF stimulation

To assess the kinetics of TPR-Ser2155 phosphorylation in response to EGF stimulation, HeLa cells were treated with EGF for increasing times and analyzed by immunoblotting using phospho-specific and total TPR antibodies (Fig. 3e). Phospho-TPR signal increased rapidly, peaking after 5–15 min and then declining, indicating a transient, stimulus-dependent response. Total TPR levels remained unchanged across all time points.

To examine whether this phosphorylation pattern is conserved across cell types, we performed similar time-course analyses in U-2 OS osteosarcoma cells (Fig. 3f) and normal BJ fibroblasts (Fig. 3g). While EGF induced Ser2155 phosphorylation in both cell types, the kinetics of the response differed from those observed in HeLa. U-2 OS cells exhibited Ser2155 phosphorylation with kinetics broadly similar to HeLa, whereas BJ fibroblasts displayed more sustained phosphorylation over time, indicating cell-type-dependent regulation of TPR Ser2155 phosphorylation in response to EGF stimulation.

Phosphorylated ERK1/2, a canonical marker of MAPK pathway activation, was monitored to assess pathway activity (Fig. 3e–g). Importantly, EGF stimulation induced TPR Ser2155 phosphorylation in all three cell types examined, and ERK activation kinetics broadly paralleled phospho-TPR dynamics across cell types, supporting a conserved, stimulus-dependent mode of TPR regulation across multiple cell types.

### EGFR inhibition suppresses EGF-induced TPR Ser2155 phosphorylation

**Fig. 4:**
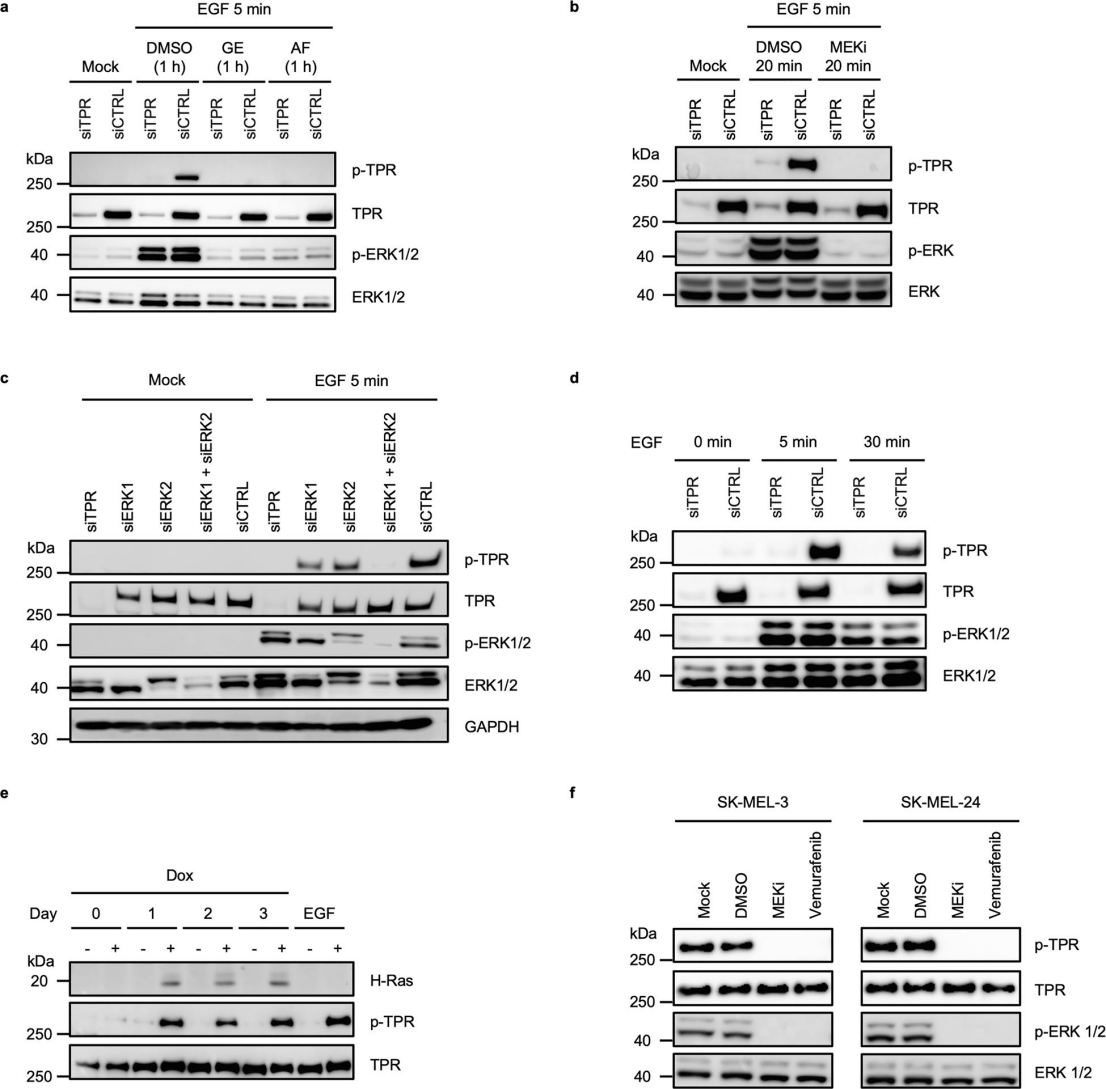
TPR phosphorylation at Ser2155 is regulated by the EGFR–MEK–ERK signaling axis and oncogenic Ras/BRAF activity. **a** Immunoblot analysis of siTPR- or siCTRL-transfected HeLa cell lysates treated with DMSO (vehicle control), gefitinib (an EGFR tyrosine kinase inhibitor), or afatinib (an EGFR tyrosine kinase inhibitor) for 1 h, followed by EGF stimulation for 5 min. Phospho-TPR-Ser2155 (p-TPR), total TPR, phospho-ERK1/2, and total ERK1/2 levels are shown. Gefitinib and afatinib effectively reduce both TPR and ERK1/2 phosphorylation, confirming that EGF-induced TPR Ser2155 phosphorylation is dependent on EGFR signaling. Data are representative of three independent experiments. **b** Immunoblot analysis of siTPR- or siCTRL-transfected HeLa cells pretreated with DMSO or U0126 (MEK inhibitor) for 20 min, followed by EGF stimulation for 5 min. U0126 significantly reduces both TPR Ser2155 and ERK1/2 phosphorylation, confirming MEK-dependent regulation. Data are representative of three independent experiments. **c** ERK1, ERK2, or both were selectively depleted in HeLa cells using siRNAs, followed by 5 min EGF stimulation. TPR phosphorylation at Ser2155 (p-TPR) is only partially reduced by single ERK1 or ERK2 knockdown but is nearly abolished by dual ERK1/2 depletion, demonstrating functional redundancy in TPR regulation. Data are representative of three independent experiments. **d** Time-course analysis of phospho-ERK1/2 and phospho-TPR-Ser2155 in HeLa cells transfected with siTPR or siCTRL and treated with EGF for 0, 5, or 30 min. TPR depletion does not impair ERK1/2 phosphorylation, confirming that TPR acts downstream of ERK1/2. Data are representative of three independent experiments. **e** Immunoblot analysis of TPR phosphorylation at Ser2155 in U-2 OS cells with doxycycline (Dox)-inducible H-RasV12 expression. Induction of H-RasV12 by Dox treatment results in robust phosphorylation of TPR at Ser2155 over time. EGF stimulation in Dox-untreated cells is included as a positive control for TPR phosphorylation via EGFR signaling. Total TPR and H-Ras levels are shown. Data are representative of three independent experiments. **f** Immunoblot analysis of phospho-TPR-Ser2155 (p-TPR), total TPR, phospho-ERK1/2, and total ERK1/2 in melanoma cell lines SK-MEL-3 and SK-MEL-24 harboring the BRAFV600E mutation. Cells were treated with vehicle control (DMSO), U0126 (MEK inhibitor) for 20 min, or vemurafenib (BRAF inhibitor) for 1 h. Data are representative of three independent experiments.

To determine whether TPR-Ser2155 phosphorylation occurs downstream of EGFR activation, HeLa cells were pretreated for 1 h with gefitinib or afatinib, two clinically approved EGFR tyrosine kinase inhibitors [38], or DMSO, before 5 min of EGF stimulation. To confirm phospho-TPR specificity, cells were transfected with siTPR or siCTRL, and lysates were analyzed by immunoblotting for phospho-TPR-Ser2155, total TPR, phospho-ERK1/2, and total ERK1/2 (Fig. 4a).

Pretreatment with either EGFR inhibitor markedly reduced EGF-induced TPR-Ser2155 phosphorylation, consistent with a requirement for EGFR kinase activity. As expected, phospho-TPR was nearly undetectable in TPR-depleted samples. ERK1/2 analysis confirmed pathway inhibition: EGF robustly activated ERK1/2 in DMSO-treated cells, whereas either inhibitor abolished ERK1/2 phosphorylation, while total ERK1/2 levels remained unaffected.

Together, these results show that EGF-induced TPR-Ser2155 phosphorylation is EGFR-dependent and coincides with ERK1/2 activation.

### MEK inhibition suppresses EGF-induced TPR-Ser2155 phosphorylation

To determine whether EGF-induced TPR-Ser2155 phosphorylation is mediated through the MEK–ERK pathway downstream of EGFR, HeLa cells were pretreated with the MEK1/2 inhibitor U0126 or DMSO for 20 min before EGF stimulation. To verify signal specificity, cells were transfected with siTPR or siCTRL, and cell lysates were analyzed by immunoblotting for phospho-TPR-Ser2155, total TPR, phospho-ERK1/2, and total ERK1/2 (Fig. 4b).

MEK1/2 inhibition completely abolished EGF-induced ERK1/2 phosphorylation, confirming effective pathway blockade. In siCTRL cells, MEK1/2 inhibition also strongly reduced EGF-induced TPR Ser2155 phosphorylation. As expected, phospho-TPR was absent in siTPR samples, validating antibody specificity. Total TPR levels were reduced only in siTPR samples, while total ERK1/2 levels remained unchanged across all conditions.

Together, these results show that EGF-induced TPR-Ser2155 phosphorylation coincides with ERK1/2 activation and is sensitive to MEK1/2 kinase inhibition.

### ERK1/2 contribute to EGF-induced TPR Ser2155 phosphorylation

To examine the role of MAPK signaling in TPR phosphorylation, ERK1, ERK2, or both kinases were selectively depleted in HeLa cells using siRNAs. After 5 min of EGF stimulation, lysates were analyzed by immunoblotting for phospho-TPR-Ser2155, total TPR, phospho-ERK1/2, and total ERK1/2 (Fig. 4c).

In siCTRL cells, EGF induced robust TPR-Ser2155 phosphorylation. Knockdown of either ERK1 or ERK2 partially reduced phospho-TPR levels, whereas combined depletion of both kinases nearly abolished the signal, consistent with contributions from both ERK1 and ERK2 to EGF-induced TPR-Ser2155 phosphorylation. As expected, ERK1/2 phosphorylation was strongly reduced in all knockdown conditions.

To test whether TPR influences ERK activation, HeLa cells were transfected with siTPR or siCTRL and stimulated with EGF for 5 or 30 min. ERK1/2 phosphorylation kinetics and magnitude were comparable in siTPR and siCTRL cells, despite loss of TPR in siTPR cells (Fig. 4d).

Together, these results show that ERK1/2 depletion reduces EGF-induced TPR-Ser2155 phosphorylation, while TPR depletion does not alter ERK1/2 activation.

### Oncogenic H-RasV12 or oncogenic BRAF induces TPR-Ser2155 phosphorylation

Activating mutations in the *RAS* oncogene result in constitutive activation of the RAS–RAF–MEK–ERK pathway [40]. To determine whether oncogenic Ras activation regulates TPR phosphorylation, we used U-2 OS cells with doxycycline-inducible expression of oncogenic H-RasV12 [41]. Cells were treated with doxycycline (Dox) for up to 3 days, and TPR-Ser2155 phosphorylation was assessed by immunoblotting (Fig. 4e). As a positive control, Dox-untreated cells were stimulated with EGF for 5 min on day 3.

In Dox-untreated cells, TPR-Ser2155 phosphorylation remained undetectable, confirming that basal Ras activity is insufficient to trigger this modification. In contrast, Dox-treated cells expressing H-RasV12 exhibited robust TPR Ser2155 phosphorylation, demonstrating that constitutive Ras activation is sufficient to drive this response.

Mutations in BRAF, a downstream effector of RAS, frequently occur in human cancers. The BRAFV600E mutation results in constitutive BRAF kinase activation and drives MEK–ERK MAPK signaling independently of upstream EGFR or RAS input [42]. SK-MEL-3 and SK-MEL-24 melanoma cell lines harbor the BRAFV600E mutation [43] and were used to examine BRAF-driven MAPK signaling.

To test whether oncogenic BRAF regulates TPR phosphorylation, SK-MEL-3 and SK-MEL-24 cells were treated with DMSO, the MEK1/2 inhibitor U0126, or the BRAFV600E-selective inhibitor vemurafenib. Lysates were analyzed for phospho-TPR-Ser2155, total TPR, phospho-ERK1/2, and total ERK1/2 (Fig. 4f). Both cell lines displayed basal ERK1/2 phosphorylation and detectable TPR Ser2155 phosphorylation, consistent with constitutive RAF–MEK–ERK MAPK pathway activation. Inhibition of either MEK1/2 or mutant BRAF markedly reduced ERK1/2 phosphorylation.

These results show that inducible expression of oncogenic H-RasV12 is sufficient to induce TPR-Ser2155 phosphorylation, and that inhibition of oncogenic BRAF reduces TPR-Ser2155 phosphorylation in relevant cancer cell models.

### Generation and molecular characterization of a *Tpr* knockout mouse model

**Fig. 5:**
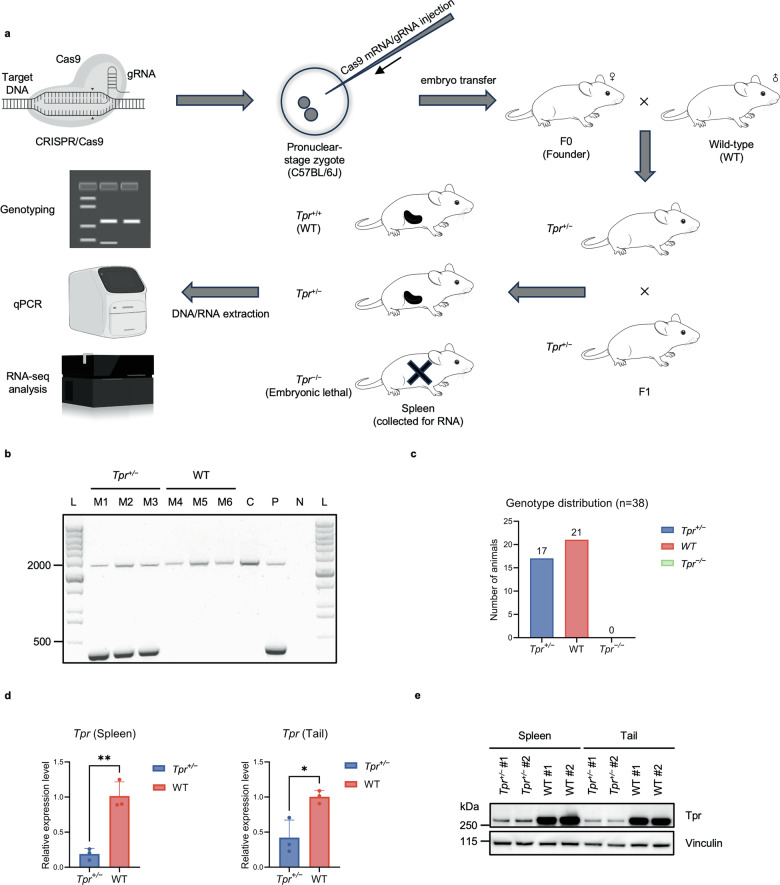
Generation and analysis of *Tpr* knockout mice. **a** CRISPR/Cas9 was microinjected into pronuclear-stage C57BL/6J zygotes, which were transferred into pseudopregnant foster mothers to generate mosaic founder mice (F0). F0 founders were outcrossed to wild-type (WT) mice to obtain F1 offspring. DNA was extracted from toe biopsies for genotyping, and spleen tissue was collected for downstream RNA-seq analysis. Intercrossing heterozygotes (*Tpr*^+/−^ × *Tpr*^+/−^) was expected to generate *Tpr*^+/+^, *Tpr*^+/−^, and *Tpr*^−/−^ genotypes; however, *Tpr*^−/−^ offspring were not recovered, indicating embryonic lethality. **b** Representative PCR genotyping of postnatal toe biopsies from heterozygous *Tpr*^+/−^ and wild-type mice. Each mouse sample is loaded in a separate lane (M1–M6). The WT allele produces a 2363-bp amplicon, whereas the targeted *Tpr* allele yields a 345-bp amplicon. WT mice show only the 2363-bp band, while *Tpr*^+/−^ mice display both PCR products. Lane C: wild-type genomic DNA control. Lane P: positive control (*Tpr*^+/−^ DNA). Lane N: no-template negative control (ddH₂O). DNA ladders (L) are shown on both sides for reference. **c** Genotype distribution of 38 offspring from *Tpr*^+/−^ intercrosses. A total of 21 WT and 17 heterozygous *Tpr*^+/−^ offspring were recovered. No homozygous knockout (*Tpr*^−/−^) mice were observed, indicating embryonic lethality. **d** Quantitative real-time PCR (qPCR) analysis of *Tpr* mRNA levels in spleen (left) and tail (right) tissues. Data are presented as mean ± SD (*n* = 3 biological replicates per condition). Statistical analysis was performed using a two-tailed unpaired *t*-test; *P < 0.05, **P < 0.01. **e** Western blot analysis of spleen and tail tissues showing reduced TPR protein levels in *Tpr*^+/−^ mice relative to WT controls, with vinculin serving as the loading control. Two biological replicates per genotype (samples #1 and #2) are shown for each tissue.

To investigate the role of TPR in vivo, we generated a CRISPR/Cas9-engineered *Tpr* knockout mouse model (Fig. 5a). A 2018-bp genomic deletion spanning exon 5 of the *Tpr-203* transcript was introduced to disrupt gene function. PCR genotyping of postnatal toe biopsies distinguished wild-type (WT) and heterozygous (*Tpr*^+/−^) alleles: WT mice showed a single 2363-bp band, whereas heterozygous mice displayed both 2363-bp and 345-bp products (Fig. 5b). No homozygous *Tpr*^−/−^ pups were recovered among the 38 genotyped offspring, indicating that TPR is required for development and/or viability (Fig. 5c).

To assess the effects of *Tpr* haploinsufficiency, we measured *Tpr* expression in spleen and tail tissues. qPCR revealed significantly reduced *Tpr* mRNA levels in *Tpr*^+/−^ mice compared to WT controls (Fig. 5d), and immunoblotting confirmed reduced TPR protein abundance in heterozygous tissues (Fig. 5e).

Collectively, these results describe the generation and initial molecular characterization of a *Tpr* knockout mouse model and document reduced *Tpr* expression at both the mRNA and protein levels in heterozygous animals.

### Altered MAPK-associated gene expression in *Tpr*^+/−^ mouse spleens

To assess the in vivo transcriptional consequences of *Tpr* haploinsufficiency, we performed RNA-seq on spleen tissues from *Tpr*^+/−^ and wild-type (WT) mice. The spleen provides a physiologically relevant context for RAS–RAF–MEK–ERK MAPK pathway activation: although EGFR expression is almost absent—particularly in the immune cell populations that typically characterize this tissue—MAPK signaling can be robustly triggered *via* B cell and T cell antigen receptors or cytokine receptors [19, 20, 44, 45].

**Fig. 6:**
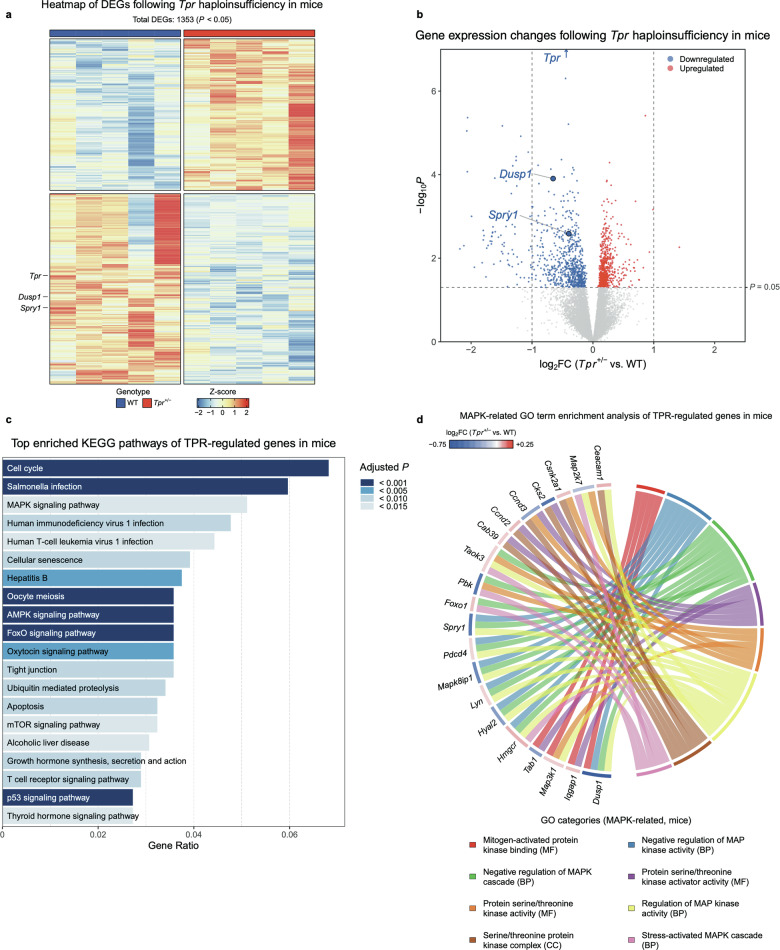
*Tpr* haploinsufficiency alters MAPK-associated gene expression in mouse spleen. **a** Heatmap of 1353 differentially expressed genes (DEGs) in spleen samples from *Tpr*^+/−^ and WT mice (*P* < 0.05). Each column represents a biological replicate (*n* = 5 mice per genotype). Hierarchical clustering reveals distinct transcriptomic profiles that separate *Tpr*^+/−^ from WT spleen samples. **b** Volcano plot showing DEGs between *Tpr*^+/−^ and WT spleens, based on statistical significance and magnitude of expression change. Significantly upregulated genes are shown in red and significantly downregulated genes are shown in blue (*P* < 0.05). Selected MAPK-associated genes are annotated. **c** KEGG pathway enrichment analysis of TPR-regulated genes in spleen, highlighting significantly enriched pathways with colors indicating adjusted *P* values. Pathways are ranked by gene ratio. The MAPK signaling pathway is among the top enriched pathways. **d** Chord diagram showing significantly enriched MAPK-related GO terms (adj. *P* < 0.05) among TPR-regulated genes in spleen. Circular nodes represent GO terms and individual genes, with connecting ribbons indicating gene–term associations. BP, biological process; CC, cellular component; MF, molecular function.

Spleens from five *Tpr*^+/−^ and five WT mice were profiled by RNA-seq. Differential expression analysis identified 1353 DEGs (*P* < 0.05), and hierarchical clustering revealed robust genotype-dependent segregation (Fig. 6a). A corresponding volcano plot (Fig. 6b) illustrates the overall distribution of differential expression, with 600 genes upregulated and 753 downregulated in *Tpr*^+/−^ spleens. As expected, *Tpr* itself was among the most strongly downregulated transcripts (*P* = 4.06e−13, log_2_FC = −0.44). Notably, *Dusp1* and *Spry1*, two well-established negative regulators of ERK/MAPK signaling [46, 47], were significantly downregulated in *Tpr*^+/−^ spleens.

KEGG pathway analysis revealed enrichment of MAPK signaling, as well as related pathways including FoxO, mTOR, and T cell receptor signaling (Fig. 6c). Gene Ontology (GO) analysis highlighted MAPK-associated categories such as “negative regulation of MAP kinase activity,” “protein serine/threonine kinase activity,” and additional MAPK-related GO categories (Fig. 6d). These GO terms reflect the distribution of DEGs across multiple MAPK-associated functional categories.

These results show that bulk spleen transcriptomes differ between *Tpr*^+/−^ and WT mice, including differential expression of genes annotated to MAPK signaling.

### Mitogen-induced *Fos* upregulation in *Tpr*^+/−^ mouse splenocytes

To functionally validate whether reduced *Tpr* levels alter MAPK-associated transcription, we examined *Fos* induction after mitogenic stimulation in cells isolated from the spleens of *Tpr*^+/−^ and WT mice. Splenocytes provide a physiologically relevant context for MAPK-dependent transcription because, for example, within splenic T cells, the RAS–RAF–MEK–ERK MAPK cascade is strongly activated by T cell receptor (TCR/CD3) engagement together with CD28 co-stimulation, independent of EGFR signaling. Since *Fos* is a downstream effector of both EGFR- and TCR-driven MAPK signaling [12, 19], this system offers an in vivo-derived platform to assess the association between reduced TPR dosage and MAPK-regulated transcription.

**Fig. 7:**
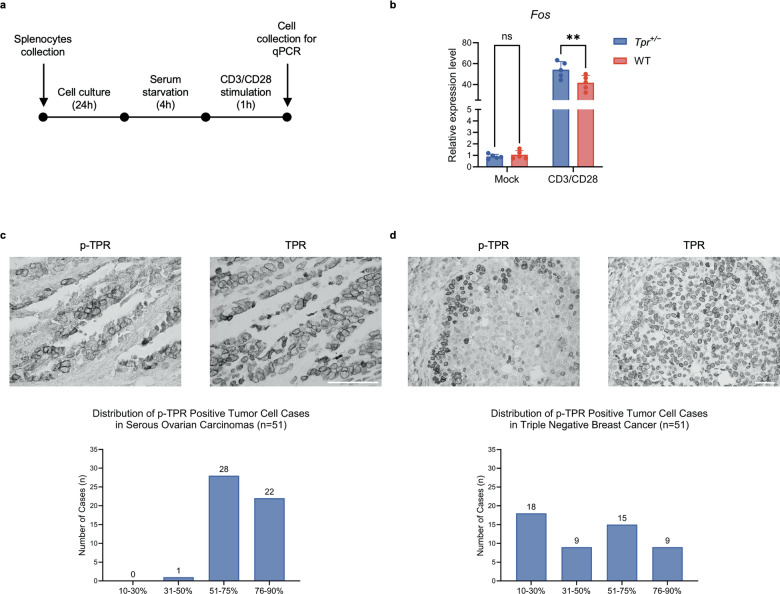
*Tpr* heterozygosity enhances CD3/CD28-induced *Fos* expression in mouse splenocytes, and p-TPR is detected in serous ovarian carcinoma and triple-negative breast cancer (TNBC). **a** Experimental timeline for anti-CD3/CD28 stimulation of splenocytes isolated from *Tpr*^+/−^ and wild-type (WT) mice. Splenocytes were cultured for 24 h, serum-starved for 4 h, and stimulated with anti-CD3 and anti-CD28 antibodies for 1 h. Cells were harvested at 29 h of culture for *Fos* mRNA qPCR analysis. **b** Quantitative real-time PCR (qPCR) analysis of *Fos* mRNA expression in splenocytes from *Tpr*^+/−^ and WT mice. CD3/CD28 stimulation significantly increased *Fos* expression in *Tpr*^+/−^ splenocytes compared to WT controls. No significant difference was observed under mock conditions. Data are presented as mean ± SD (*n* = 5 mice per genotype). Statistical analysis was performed using two-way ANOVA followed by Šídák’s multiple-comparisons test; ns, not significant; **P < 0.01. **c** Immunohistochemical analysis of phospho-TPR-Ser2155-positive tumor cells in 51 cases of serous ovarian carcinoma. The majority of cases exhibited high levels of phospho-TPR-Ser2155 positivity, with 54.9% of samples in the 51–75% range, 43.1% in the 76–90% range, and 2.0% in the 31–50% range. Quantification of phospho-TPR-Ser2155-positive tumor cells was performed by analyzing immunohistochemically stained sections, and data are presented as percentage categories. **d** Immunohistochemical analysis of phospho-TPR-Ser2155-positive tumor cells in 51 cases of TNBC. A diverse distribution was observed, with 35.3% of cases showing 10–30% positivity, 17.6% showing 31–50%, 29.4% in the 51–75% range, and 17.6% in the 76–90% range. Quantification of phospho-TPR-Ser2155-positive tumor cells was performed by analyzing immunohistochemically stained sections, and data are presented as percentage categories.

Splenocytes were cultured, serum-starved, and stimulated for 1 h with anti-CD3 and anti-CD28 antibodies to engage their respective receptors (Fig. 7a). qPCR analysis showed low basal *Fos* expression in both genotypes, and robust induction upon stimulation, with *Tpr*^+/−^ splenocytes exhibiting significantly higher *Fos* mRNA levels than WT controls (Fig. 7b).

These findings show that mitogenic stimulation induces higher *Fos* mRNA levels in *Tpr*^+/−^ splenocytes than in WT controls.

### TPR phosphorylation patterns in human serous ovarian carcinoma and TNBC

To evaluate the clinical relevance of TPR phosphorylation, we performed immunohistochemical (IHC) analysis of phosphorylated TPR-Ser2155 (p-TPR) in cohorts of serous ovarian carcinomas and triple-negative breast cancer (TNBC), two tumor types frequently exhibiting dysregulated EGFR–RAS–RAF–MEK–ERK MAPK signaling [48, 49]. For each cohort, p-TPR positivity was quantified as the percentage of stained tumor cells across 51 cases.

In serous ovarian carcinoma, p-TPR staining was almost uniformly high, as 54.9% of cases showed positivity in 51–75% of tumor cells, and 43.1% showed positivity in 76–90% (Fig. 7c).

In contrast, TNBC samples displayed greater heterogeneity. The largest subset (35.3%) showed 10–30% p-TPR–positive cells; 17.6% of cases showed 31–50% p-TPR–positive cells; and 29.4% and 17.6% of cases showed 51–75% and 76–90% p-TPR–positive cells, respectively (Fig. 7d).

Together, these results show that TPR-Ser2155 phosphorylation occurs commonly in human tumors and that the staining patterns differ between serous ovarian carcinoma and TNBC.

## Discussion

The EGFR–RAS–RAF–MEK–ERK MAPK cascade is a central regulator of cell proliferation, differentiation, and survival and is frequently aberrantly activated in human cancers [12, 13]. Activation of EGFR at the plasma membrane initiates a signaling cascade culminating in induction of immediate-early genes like *FOS* and *JUN*, which promote mitogenic transcriptional programs and cell-cycle entry [50]. Despite extensive characterization of cytoplasmic signaling events, how mitogenic signals are transmitted to nuclear transcriptional machinery with appropriate timing, amplitude, and fidelity remains incompletely understood. In addition to their established role in nucleocytoplasmic transport, components of the nuclear pore complex (NPC) have been increasingly implicated in chromatin organization, gene expression, and the prevention of transcription-associated genomic instability [8–10], raising the possibility that NPC-associated factors contribute directly to signal-dependent transcription.

Prior quantitative phosphoproteomic studies identified the nucleoporin TPR as an EGF-responsive phosphorylation target [15, 16], consistent with studies suggesting that TPR can interact with and be phosphorylated by ERK1/2 kinases [17, 18]. However, the functional relevance of TPR regulation by MAPK signaling and its contribution to mitogen-induced transcriptional responses had remained unclear. In this study, we combine transcriptomic analyses with biochemical, genetic, and in vivo approaches to support a model in which TPR functions as a MAPK-responsive nuclear component that modulates mitogen-induced transcriptional programs.

A major technical barrier to studying TPR regulation has been the lack of reagents capable of detecting endogenous phosphorylated TPR. To overcome this limitation, we generated and validated a phospho-specific monoclonal antibody recognizing TPR phosphorylated at Ser2155. This antibody enables robust detection of endogenous phospho-TPR by immunoblotting, immunofluorescence, and immunohistochemistry. Using this tool, we demonstrate that TPR-Ser2155 phosphorylation is rapidly and transiently induced following EGF stimulation and is conserved across both cancerous (HeLa, U-2 OS) and non-cancerous (BJ) human cell types. ERK activation kinetics closely mirrored phospho-TPR-Ser2155 dynamics, supporting the notion that Ser2155 phosphorylation occurs early during mitogenic signaling and is regulated by MAPK pathway activity. These observations are consistent with, and extend, previous phosphoproteomic findings reporting EGF-induced TPR-Ser2155 phosphorylation in HeLa cells [15, 16], reinforcing the concept that NPC components are dynamic signaling-responsive targets rather than static structural elements.

Pharmacological and genetic perturbations further position TPR-Ser2155 phosphorylation within the canonical EGFR–MAPK cascade. Inhibition of EGFR kinase activity using gefitinib or afatinib suppressed EGF-induced TPR-Ser2155 phosphorylation in parallel with loss of ERK1/2 activation, indicating dependence on upstream EGFR signaling. While EGFR activation triggers this modification, these data do not exclude the possibility that Ser2155 phosphorylation may also be regulated downstream of other mitogenic pathways. Consistent with canonical MAPK signaling, inhibition of MEK1/2 with the U0126 inhibitor abolished ERK1/2 activation and markedly reduced Ser2155 phosphorylation. Together, these results indicate that EGF-induced TPR-Ser2155 phosphorylation depends on MEK–ERK signaling downstream of EGFR.

Depletion of ERK1 or ERK2 partially reduced TPR phosphorylation, whereas combined ERK1/2 depletion nearly abolished the phospho-TPR-Ser2155 signal, demonstrating that both ERK1 and ERK2 contribute to phosphorylation of TPR-Ser2155. Importantly, TPR depletion did not affect ERK1/2 activation kinetics or magnitude, indicating that TPR functions downstream of ERK along the MAPK signaling axis, consistent with reports identifying TPR as an ERK substrate and interaction partner [17, 18].

Beyond ligand-induced EGFR signaling, our data demonstrate that TPR-Ser2155 phosphorylation also reflects oncogenic activation of the MAPK pathway. Inducible expression of oncogenic H-RasV12 was sufficient to induce robust TPR-Ser2155 phosphorylation in the absence of external growth factors, supporting regulation downstream of RAS–MAPK signaling. Similarly, melanoma cell lines harboring the oncogenic BRAFV600E mutation displayed constitutive TPR-Ser2155 phosphorylation that was reduced upon inhibition of mutant BRAF. Combined with the EGF-driven responses, these findings support a model in which TPR-Ser2155 phosphorylation is mediated by activation of the RAS–RAF–MEK–ERK MAPK pathway in response to either physiological receptor stimulation or oncogenic mutations in upstream pathway components. How TPR-Ser2155 phosphorylation mechanistically alters TPR function—potentially by modulating TPR’s interactions with chromatin-associated or transcriptional regulators—and whether the transcriptional effects described here are directly dependent on this specific modification or reflect broader MAPK-mediated regulation of TPR, remain important questions for future investigations.

To determine whether MAPK-dependent regulation of TPR is accompanied by functional consequences for transcriptional programs, we performed RNA-seq analyses in TPR-depleted HeLa cells under mock-treated and EGF-stimulated conditions. These experiments revealed that TPR contributes to transcriptional regulation in a context-dependent manner, with distinct and overlapping gene sets affected in unstimulated and mitogen-stimulated cells. Many of the differentially expressed genes are associated with MAPK signaling and are normally regulated by EGF, suggesting that TPR modulates transcriptional outputs across signaling states. Importantly, previous studies have shown that TPR is dispensable for NPC assembly and its depletion does not disrupt the localization of other nuclear basket components such as Nup153 and Nup98 [51, 52], indicating that the transcriptional changes observed here are unlikely to reflect global disruption of NPC architecture and instead point to specific regulatory functions of TPR.

A prominent feature of the transcriptomic data is altered expression of MAPK-associated genes and regulators. Under mock-treated conditions, TPR depletion increased expression of multiple MAPK-linked genes, including *JUN*, *EREG*, *CXCL1*, *DUSP10*, *NOX4*, *GDF15*, and *DKK1*, consistent with altered baseline signaling or feedback regulation. Across both mock-treated and EGF-stimulated conditions, a shared subset of genes—including *FOXQ1*, *RHOU*, *ONECUT2*, *FAM83B*, *IGF1*, and *RBM15*—was sensitive to TPR depletion, suggesting that TPR contributes to maintaining transcriptional programs across signaling contexts.

Following EGF stimulation, additional MAPK-associated nodes were affected, including increased *FOS* and *SOX4* and reduced expression of the negative regulator *SPRY4*, indicating that TPR loss impacts both positive and negative components of MAPK-responsive transcriptional circuitry.

Among these transcriptional changes, *FOS* emerged as a particularly sensitive stimulus-dependent output modulated by TPR. While *FOS* expression was not altered under mock-treated conditions, it was robustly upregulated following EGF stimulation in TPR-depleted HeLa compared with control cells, as confirmed by qPCR. This enhanced induction was suppressed by gefitinib, indicating dependence on EGFR kinase activity.

To determine whether the altered transcriptional response caused by TPR depletion was accompanied by cellular changes in mitogenic output, we assessed EGF-driven cell-cycle progression and observed a shift in cell-cycle distribution, with TPR-depleted cells showing a higher proportion of G1-phase cells following EGF stimulation. Short-term TPR depletion did not alter cell-cycle distribution in unstimulated cells, consistent with prior reports [10], confirming that the observed phenotype arises specifically during the mitogenic response. Although *FOS* is hyper-induced in TPR-depleted cells, this is unlikely to directly explain the altered G1-phase response, as c-Fos typically promotes the G1–S transition [12]. Instead, the uncoupling of immediate-early gene induction from cell-cycle progression points to a broader disruption of signaling fidelity in MAPK-dependent transcriptional programs, with likely complex consequences for cell proliferation control.

To extend our analyses to in vivo settings, we generated a CRISPR/Cas9-engineered *Tpr* knockout mouse model. The absence of viable homozygous knockout (*Tpr*^−/−^) animals indicates an essential requirement for TPR in development and/or viability. Accordingly, our in vivo analyses focus on *Tpr* haploinsufficiency, which allows assessment of MAPK-dependent transcriptional regulation in murine tissues without the confounding effects of developmental lethality. In *Tpr*^+/−^ spleens, our bulk-tissue RNA-seq revealed widespread transcriptomic alterations with enrichment of MAPK-related pathways, including downregulation of MAPK negative regulators such as *Dusp1* and *Spry1*. The distribution of differentially expressed genes across multiple MAPK-associated categories indicates that *Tpr* haploinsufficiency is associated with transcriptomic changes at several nodes of the MAPK signaling network, consistent with observations in TPR-depleted HeLa cells. Functionally, stimulated splenocytes from *Tpr*^+/−^ animals displayed enhanced *Fos* induction following CD3/CD28 engagement. Despite engaging distinct upstream receptors—EGFR in epithelial cells and TCR in splenocyte cultures—both systems converge on MAPK activation, consistent with a conserved association between reduced TPR levels and altered MAPK-linked transcriptomic responses across these physiological contexts.

Finally, we examined whether phospho-TPR-Ser2155 is detectable in human tumors known to feature MAPK pathway dysregulation. Immunohistochemical analyses revealed consistently high phospho-TPR-Ser2155 levels in serous ovarian carcinoma and more heterogeneous staining patterns in triple-negative breast cancer (TNBC). EGFR overexpression has been reported in up to 60% of epithelial ovarian cancers [48], consistent with the uniformly high p-TPR levels observed. In contrast, mutant Ras is infrequent, and EGFR overexpression in TNBC commonly occurs in a lower proportion of cases and is associated with heterogeneous pathway activation [49]. Our data suggest that p-TPR-Ser2155 may serve as a functional readout of downstream MAPK pathway activation in these tumors and could potentially provide a biomarker to help guide treatment with clinically used kinase inhibitors in the future.

At the conceptual cell biology level, our results expand current models of MAPK signaling by implicating nuclear pore complex components as active participants in signal-dependent transcription and identify TPR as a key factor shaping mitogen-induced transcriptional outputs.

These findings provide a framework for future studies examining how deregulation of TPR, or its MAPK-dependent phosphorylation, contributes to disease-relevant processes across diverse cell and tissue types, including cancer and immune cells such as T and B lymphocytes, where MAPK signaling plays central roles in proliferation, differentiation, and functional activation.

## Methods

### Cell culture and treatments

HeLa cells (European Collection of Authenticated Cell Cultures), U-2 OS cells (ATCC), BJ cells (Cell Bank/Stem Cell Bank, Chinese Academy of Sciences), and U-2 OS H-RasV12 cells (generated in house from parental U-2 OS cells) were maintained in Dulbecco’s Modified Eagle’s Medium (DMEM) (Sigma-Aldrich, #D5671) supplemented with 10% fetal bovine serum (FBS) (Vistech, #SE100-B), 1 mM sodium pyruvate (Gibco, #11360070), 1× MEM Non-Essential Amino Acids (Gibco, #11140050), 2 mM GlutaMAX (Gibco, #35050061), and 100 U/mL and 100 μg/mL penicillin-streptomycin (Gibco, #15140122). H-RasV12 expression was induced with 2 μg/mL doxycycline (Sigma-Aldrich, #D9891) for the indicated duration. SK-MEL-3 and SK-MEL-24 melanoma cell lines (both from ATCC) were cultured in RPMI 1640 medium (Gibco, #21875034) supplemented with 10% FBS (Sigma-Aldrich, #F7524), and 100 U/mL and 100 μg/mL penicillin-streptomycin (Gibco, #15140122). All cell lines were maintained in a humidified incubator at 37 °C with 5% CO₂. Mouse splenocytes were cultured in RPMI 1640 medium (Gibco, #11875093) supplemented with 10% heat-inactivated fetal bovine serum (FBS) (Vazyme, #F101-01), 2 mM GlutaMAX (Gibco, #35050061), 100 U/mL penicillin and 100 μg/mL streptomycin (Gibco, #15140122), and 0.5 mM 2-mercaptoethanol (Gibco, #31350010).

For treatments, cells were stimulated with human EGF (Gibco, #PHG6045, 150 ng/mL), U0126-EtOH (Selleck Chemicals, #S1102, 10 μM), Afatinib (BIBW2992) (Selleck Chemicals, #S1011, 5 μM), Gefitinib (ZD1839) (Selleck Chemicals, #S1025, 5 μM), or Vemurafenib (PLX4032) (Selleck Chemicals, #S1267, 3 μM) at the specified final concentrations. Vehicle controls, including DMSO or ethanol, were used as appropriate. Cells were treated for the indicated durations before analysis. All cell lines used in this study were routinely tested for mycoplasma contamination using PCR-based assays and were consistently confirmed to be mycoplasma-negative.

### Monoclonal antibody generation and validation

A monoclonal antibody targeting phosphorylated TPR at Ser2155 (p-TPR) was generated using hybridoma technology. A synthetic phosphorylated peptide corresponding to the Ser2155 region of the TPR protein was conjugated to keyhole limpet hemocyanin (KLH) and used for immunization. Three Balb/c and three C57BL/6 mice were immunized following a conventional prime-boost strategy, and antiserum was collected after the third immunization for preliminary screening. Hybridoma generation was performed by fusing splenocytes from immunized mice with SP2/0 myeloma cells. Clones were screened by indirect ELISA, first against the phosphorylated peptide and subsequently counter-screened with the non-phosphorylated counterpart to ensure specificity. Positive hybridomas were subcloned by limiting dilution, expanded, and further validated for phospho-specificity. The selected hybridoma clone was expanded in roller bottle culture, and monoclonal antibodies were purified via protein G affinity chromatography. Antibody specificity was validated using Western blot, immunofluorescence, and immunohistochemistry in TPR-depleted cells. Additionally, a peptide blocking assay was performed to confirm the phospho-specificity of the antibody.

### Peptide blocking assay for phospho-TPR Ser2155 antibody validation

To assess the specificity of the phospho-TPR Ser2155 antibody, a peptide blocking assay was performed using phosphorylated (pS2155) and non-phosphorylated (S2155) peptides as competitive inhibitors. Peptides were reconstituted in UltraPure™ DNase/RNase-Free Distilled Water (Invitrogen, #10977035) to a final concentration of 1.25 μg/μL. 9.17 μg of phospho-TPR Ser2155 antibody was diluted in 10 mL of 1× PBS with 0.01% Tween-20 and 1% milk, then pre-incubated with either the phosphorylated or non-phosphorylated peptide at a 5:1 (peptide:antibody) mass ratio for 90 min at room temperature. Following incubation, the antibody-peptide mixtures were applied to blocked Western blot membranes containing whole-cell lysates from EGF-treated and untreated HeLa cells, as well as siRNA-mediated TPR-depleted and control HeLa cells. Membranes were incubated overnight at 4 °C, washed, and then incubated with HRP-conjugated secondary antibody. Signal detection was performed using FG Super Sensitive ECL Luminescence Reagent (Meilunbio, #MA0186). The peptide blocking assay was also used in immunofluorescence (IF) and immunoperoxidase staining (IHC) experiments to validate antibody specificity in fixed-cell and tissue-based applications. In these assays, antibody pre-incubation with blocking peptides was performed before applying the antibody to samples, and signal intensity was compared between blocked and unblocked conditions to assess specificity.

### Transfections

siRNA transfections were performed using Lipofectamine RNAiMAX Transfection Reagent (Invitrogen, #13778075) according to the manufacturer’s instructions. siRNAs were used at a final concentration of 25 nM, and all assays were conducted 72 h post-transfection unless stated otherwise. The specific oligonucleotides used for each assay in this study are listed in Supplementary Table 1.

### Immunoblotting

Whole-cell extracts were prepared using 1× Laemmli sample buffer (2% SDS, 10% glycerol, 50 mM Tris-HCl, pH 6.8). Cell lysates were sonicated (Xiaomei Chaosheng Instruments, XM-650T, China) and centrifuged at 16,000 × *g* at 4 °C for 30 min. Protein concentration was determined using the BCA protein assay (Thermo Fisher Scientific, #23225). Proteins were resolved by SDS-PAGE and transferred onto PVDF membranes (Millipore, #IPFL00010). Membranes were blocked in 1× PBS with 0.01% Tween-20 containing 5% milk powder at room temperature for 30 min. Primary antibodies were diluted in 1× PBS with 0.01% Tween-20 and 1% milk and incubated overnight. Secondary antibodies were diluted in the same buffer and incubated for 1 h at room temperature before detection using FG Super Sensitive ECL Luminescence Reagent (Meilunbio, #MA0186). The following primary antibodies were used: c-H-Ras (Ab-1) (Sigma-Aldrich, #OP23, mouse, 1:1000), ERK1/ERK2 (Abclonal, #A10613, mouse, 1:2000), ERK1/ERK2 (Abclonal, #A4782, rabbit, 1:2000), Phospho-p44/42 MAPK (ERK1/2) (Thr202/Tyr204) (Cell Signaling Technology, #4370, rabbit, 1:1000), Phospho-ERK1 (T202 + Y204) + ERK2 (T185 + Y187) (Huabio, #ET1610-13, rabbit, 1:2500), GAPDH (Servicebio, #GB15002, mouse, 1:10000), TPR total (Sigma-Aldrich, #HPA019661, rabbit, 1:1000), p-TPR Ser2155 (developed in-house, mouse, 1:1000), Vinculin (Proteintech, #66305-2-Ig, mouse, 1:1000).

The following secondary antibodies were used: goat anti-mouse IgG H&L (HRP) (Abcam, #ab205719, goat, 1:5000), goat anti-rabbit IgG H&L (HRP) (Abcam, #ab205718, goat, 1:5000).

### Immunostaining

Cells growing on 9 mm coverslips were fixed with 4% formaldehyde (Sinopharm Chemical Reagent, #10010061) in 1× PBS for 15 min at room temperature, then washed twice with 1× PBS. Permeabilization was performed at room temperature for 15 min using 0.2% Triton X-100 (Beyotime, #ST795) in 1× PBS. Coverslips were then washed twice with 1× PBS and blocked with 1× PBS containing filtered 3% BSA (Beyotime, #ST023) and 0.1% Triton X-100 (Beyotime, #ST795) at room temperature for 30 min. Primary and secondary antibodies were diluted in 1× PBS containing filtered 3% BSA (Beyotime, #ST023) and 0.1% Triton X-100 (Beyotime, #ST795). Coverslips were incubated with primary antibodies at room temperature for 1 h, followed by three 5-min washes with 1× PBS.

Coverslips were then incubated with secondary antibodies at room temperature for 1 h, washed once with 1× PBS for 5 min, stained with DAPI (Beyotime, #C1005) for 5 min, washed twice with 1× PBS for 5 min, and finally mounted using 2 μL of antifade mounting medium (Beyotime, #P0126).

The following primary antibodies were used: TPR total (Sigma-Aldrich, #HPA019661, rabbit, 1:500), TPR total (Abcam, #ab84516, rabbit, 1:500), p-TPR Ser2155 (developed in-house, mouse, 1:1000).

The following secondary antibodies were used: Alexa Fluor 488-labeled Goat Anti-Mouse IgG (H + L) (Beyotime, #A0428, goat, 1:500), Alexa Fluor 555-labeled Donkey Anti-Rabbit IgG (H + L) (Beyotime, #A0453, donkey, 1:500).

### Microscopy

Fluorescence images were acquired using a Nikon Eclipse Ni-U upright microscope (Nikon, Japan) equipped with a 20×/0.50 NA air objective. Fluorescence imaging was performed using specific excitation/emission filters for DAPI (Ex: 361–389 nm/Em: 430–490 nm), Alexa Fluor 488 (Ex: 450–490 nm/Em: 506.5–561.5 nm), and Alexa Fluor 555 (Ex: 525–575 nm/Em: 592.5–667.5 nm). Images were captured using a Nikon DS-Ri2 camera (Nikon, Japan) and analyzed with NIS-Elements software (version 5.11.01, Nikon, Japan). Exposure times were optimized and kept constant across all samples to ensure comparability.

### Confocal microscope

Confocal fluorescence images were acquired using a Zeiss LSM880 confocal microscope (Zeiss, Germany) equipped with a 40×/1.40 NA oil-immersion objective. Fluorescence imaging was performed using the following excitation/emission settings: DAPI (Ex 405 nm/Em 410–507 nm), Alexa Fluor 488 (Ex 488 nm/Em 493–572 nm), and Alexa Fluor 555 (Ex 561 nm/Em 566–697 nm). Images were analyzed using ZEN 2.3 SP1 FP3 software (version 14.0.27.201, Zeiss, Germany). Scanning parameters were optimized and kept constant across all samples to ensure comparability.

### RNA-seq sample preparation from HeLa cells and mouse spleen tissue

Total RNA from HeLa cells was extracted using the Super FastPure Cell RNA Isolation Kit (Vazyme, #RC102), following the manufacturer’s instructions. Total RNA from mouse spleen tissue was extracted using TRNzol Universal Reagent (Tiangen Biotech, #DP424) by Novogene, also according to the manufacturer’s protocol. RNA integrity was assessed using the Bioanalyzer 2100 system (Agilent Technologies, CA, USA) prior to library construction. RNA-seq libraries were prepared using the Fast RNA-seq Lib Prep Kit V2 (ABclonal, #RK20306), and high-throughput sequencing was performed on the Illumina NovaSeq X Plus platform. All library preparation and sequencing procedures were carried out by Novogene.

### RNA-seq analysis of HeLa cells

Quality control and trimming of raw sequencing reads were conducted using FastQC (version 0.12.1) and Trimmomatic (version 0.39) [53], respectively. The GRCh38.p14 (hg38) reference genome and corresponding gene annotation files were obtained from NCBI (https://www.ncbi.nlm.nih.gov/datasets/genome/GCF_000001405.40/). The reference genome index was built using HISAT2 (version 2.2.0) [54], and paired-end clean reads were aligned to the genome using the same software.

For gene expression quantification, the Rsubread package (version 2.20.0) [55] and its featureCounts function were used to calculate read counts mapped to each gene. Differential expression analysis across four comparison groups was performed using the DESeq2 R package (version 1.46.0) [56]. Genes with a Benjamini–Hochberg (BH) adjusted *P* value < 0.05 and |log_2_ fold change (log_2_FC)| > 1 were considered significantly differentially expressed. The resulting DEGs were visualized using Venn diagrams generated by the VennDiagram R package (version 1.7.3). A principal component analysis (PCA) plot and volcano plots were generated using the ggplot2 R package (version 3.5.1). Clustered heatmaps were generated using the ComplexHeatmap R package (version 2.22.0) on variance-stabilizing transformation (VST) values and scaled by row [57]. Gene expression analysis for individual transcripts was performed using DESeq2-normalized counts, and boxplots display log₂-transformed normalized values. Gene Ontology (GO) [58, 59] and Kyoto Encyclopedia of Genes and Genomes (KEGG) [60] pathway enrichment analyses of DEGs (adjusted *P* value < 0.05 and |log_2_ fold change (log_2_FC)| > 0.3) were performed using the Over-Representation Analysis (ORA) method, implemented in the clusterProfiler R package (version 4.14.4) [61]. Gene Set Enrichment Analysis (GSEA) [62, 63] for both GO and KEGG datasets was also performed with clusterProfiler. Statistical significance was assessed using the Benjamini–Hochberg (BH) method for multiple testing correction, with an adjusted *P* value threshold of 0.05 to control the false discovery rate (FDR). Visualization of enrichment results included bar plots generated with the ggplot2 R package (version 3.5.1) and chord diagrams created using the circlize R package (version 0.4.16) [64].

### RNA-seq analysis of mouse spleen tissue

Raw sequencing data in FASTQ format were processed using fastp (version 0.23.2) to obtain high-quality clean reads [65]. The mouse reference genome (GRCm38/mm10) and corresponding gene annotation files were downloaded from NCBI [66]. Genome indexing was performed using HISAT2 (version 2.2.5) and paired-end reads were aligned to the reference genome using the same tool. Gene-level read counts were generated using featureCounts (version 2.0.1) [67]. Differential expression analysis was conducted using the DESeq2 R package (version 1.46.0), and genes with *P* value < 0.05 were considered significantly differentially expressed. Variance-stabilizing transformation (VST) values were corrected for batch effects using the removeBatchEffect function from the limma R package (version 3.62.1) [56, 68] and subsequently scaled by row for heatmap generation. Gene Ontology (GO) [58, 59] and Kyoto Encyclopedia of Genes and Genomes (KEGG) [60] pathway enrichment analyses were performed using the Over-Representation Analysis (ORA) method implemented in the clusterProfiler R package (version 4.14.4). Statistical significance was assessed using the Benjamini–Hochberg (BH) method for multiple testing correction, with an adjusted *P* value threshold of 0.05 to control the false discovery rate (FDR). Visualizations included volcano plots and bar plots generated with ggplot2 R package (version 3.5.1), clustered heatmaps produced using ComplexHeatmap (version 2.22.0), and chord diagrams created with circlize (version 0.4.16). All downstream analyses and visualizations were carried out in the R environment (version 4.4.2; R Core Team, 2024).

### Quantitative real-time PCR analysis of HeLa cells and mouse splenocytes

Total RNA was extracted from HeLa cells using the Super FastPure Cell RNA Isolation Kit (Vazyme, #RC102) according to the manufacturer’s instructions. Reverse transcription was performed using the HiScript IV All-in-One Ultra RT SuperMix for qPCR (Vazyme, #R433). Quantitative real-time PCR (qPCR) was carried out using the ChamQ Blue Universal SYBR qPCR Master Mix (Vazyme, #Q312) on a CFX96 Touch Real-Time PCR Detection System (Bio-Rad, USA).

Relative transcript levels were quantified using the 2^-ΔΔCt method [69], with normalization to *ACTB* or *GAPDH* for human samples and *Gapdh* for mouse samples. Data are presented as mean ± standard deviation (SD) from at least three biologically independent samples or animals per condition. Visualizations were generated in GraphPad Prism 9 (GraphPad Software) as bar plots with overlaid data points. The primers used in this study are listed in Supplementary Table 1.

### Generation and maintenance of *Tpr* knockout mice

*Tpr* knockout (*Tpr*-KO) mice (Strain No. T014478) were obtained from GemPharmatech (Nanjing, China). The mice were generated using CRISPR/Cas9-mediated genome editing to delete the 2018 bp region spanning exon 5 of the *Tpr-203* transcript (ENSMUST00000124973.8), resulting in a disruption of protein function. The strain, designated C57BL/6JGpt-*Tpr*^*em1Cd2018*^/Gpt, was maintained on a C57BL/6J genetic background and housed under specific pathogen-free (SPF) conditions. Heterozygous *Tpr*^+/−^ mice were viable and used for downstream phenotypic and molecular analyses, while no homozygous knockout (*Tpr*^−/−^) animals were obtained.

### Genotyping of *Tpr* knockout mice

Genotyping was performed using genomic DNA isolated from toe biopsies at postnatal day 5 (P5). DNA was extracted using the One Step Mouse Genotyping Kit (Vazyme, #PD101-01) according to the manufacturer’s instructions. Primers were designed by GemPharmatech (Nanjing, China) and synthesized by Tsingke Biotechnology (Beijing, China). PCR amplification of the targeted genomic region yielded a 2363 bp product for the wild-type (WT) allele and a 345 bp product corresponding to the knockout allele. Heterozygous (*Tpr*^+/−^) mice exhibited both bands, confirming the presence of one intact and one disrupted allele. Genotyping outcomes were categorized as WT or heterozygous (*Tpr*^+/−^); no homozygous knockout (*Tpr*^−/−^) animals were recovered.

### Isolation and culture of mouse splenocytes

Spleens were aseptically isolated from euthanized mice, and surrounding tissues, including adipose, were removed. Each spleen was transferred into a 50 mL centrifuge tube containing 5 mL RPMI 1640 medium (Gibco, #11875093) supplemented with 10% heat-inactivated fetal bovine serum (FBS) (Vazyme, #F101-01), 2 mM GlutaMAX (Gibco, #35050061), 100 U/mL penicillin and 100 μg/mL streptomycin (Gibco, #15140122), and 0.5 mM 2-mercaptoethanol (Gibco, #31350010). Spleens were gently homogenized using the flat end of a 1 mL sterile syringe plunger on a 70 μm sterile cell strainer (Biosharp, #BS-70-CS) placed over a Petri dish. The resulting cell suspension was filtered through the strainer into a 50 mL Falcon tube. The dish was washed with 5 mL 1× PBS (Beyotime, #C0221B), and the wash was filtered through the same strainer into the same tube. Cells were pelleted by centrifugation at 500 × *g* for 8 min at room temperature. The supernatant was discarded, and the pellet was resuspended in 1 mL red blood cell lysis buffer (Roche, #11814389001) and incubated for 2 min at room temperature. Lysis was stopped by adding 4 mL RPMI 1640 medium, followed by centrifugation at 500 × *g* for 8 min. The supernatant was removed, and the pellet was resuspended in 10 mL fresh RPMI 1640 medium. To remove residual clumps or debris, the cell suspension was passed through a 70 μm cell strainer and transferred into a clean 50 mL tube for subsequent culture and stimulation experiments.

### Mouse splenocyte activation

To assess *Fos* mRNA expression in response to T-cell receptor (TCR) stimulation, freshly isolated mouse splenocytes were resuspended in RPMI 1640 medium (Gibco, #11875093) supplemented with 10% heat-inactivated fetal bovine serum (FBS) (Vazyme, #F101-01), 2 mM GlutaMAX (Gibco, #35050061), 100 U/mL penicillin and 100 μg/mL streptomycin (Gibco, #15140122), 0.5 mM 2-mercaptoethanol (Gibco, #31350010), and cultured for 24 h.

Cell number and viability were evaluated using Trypan blue exclusion, and cells were adjusted to a final concentration of 3 × 10⁶ cells/mL. A total of 4 mL of the cell suspension was seeded into each well of a 6-well plate (Vazyme, #CCP01006-B) in RPMI 1640 medium without fetal bovine serum (FBS) to induce serum starvation for 4 h. Following starvation, cells were stimulated for 1 h with anti-CD3e monoclonal antibody (clone 145-2C11, Thermo Fisher Scientific, #16-0031-82) and anti-CD28 monoclonal antibody (clone 37.51, Thermo Fisher Scientific, #16-0281-82), each at a final concentration of 2 μg/mL. RNA was extracted using the Super FastPure Cell RNA Isolation Kit (Vazyme, #RC102-01) according to the manufacturer’s instructions following stimulation. *Fos* mRNA expression was then analyzed.

### Flow cytometry

HeLa cells were harvested by trypsinization, washed with 1× PBS, and fixed in 2% formaldehyde (Sinopharm Chemical Reagent, #10010061) for 20 min on ice. Following fixation, cells were washed with 1x PBS containing filtered 1% BSA (Beyotime, #ST023) and treated with cold ethanol (−20 °C; Sinopharm Chemical Reagent, #10009218) for 30 min on ice. After ethanol treatment, cells were washed again with filtered 1% BSA in 1× PBS and incubated with propidium iodide (PI; Sangon Biotech, #A601112) at a final concentration of 2.5 µg/mL in 1× PBS in the presence of RNase A (Solarbio, #R1030) at 250 µg/mL. A PI-negative sample was included as a staining control. Samples were acquired using an ACEA NovoCyte cytometer (ACEA Biosciences, Agilent, USA) and analyzed with NovoExpress software (version 1.6.2).

### Immunohistochemical analysis

Five-micrometer tissue sections were cut from formalin-fixed, paraffin-embedded tissue blocks and mounted on Super Frost Plus slides (Menzel-Gläser, Braunschweig, Germany). Slides were baked at 60 °C for 60 min, deparaffinized, and rehydrated through graded alcohol rinses. Heat-induced antigen retrieval was performed by immersing the slides in citrate buffer (pH 6.0) and heating them in a microwave oven for 15 min. Immunohistochemistry staining was then performed using the following primary antibodies: TPR antibody (Sigma-Aldrich, #HPA019661, rabbit, 1:1000) and p-TPR-Ser2155 (developed in-house, mouse, 1:1000). The sections were incubated with the primary antibodies overnight in a cold room, followed by processing using the indirect streptavidin-biotin-peroxidase method with the Vectastain Elite kit (Vector Laboratories, Burlingame, CA, USA) and nickel-sulfate-based chromogen enhancement detection as previously described, without nuclear counterstaining [70]. Negative controls were performed by incubating sections with nonimmune sera. The results were evaluated by two experienced researchers, including a senior oncopathologist, and scored into four categories based on the percentage of p-TPR Ser2155-positive cells: (i) 10–30%, (ii) 31–50%, (iii) 51–75%, and (iv) 76–90%. The cohort of archival formalin-fixed, paraffin-embedded tissues from human triple-negative breast carcinomas (*n* = 51) was described recently [71]. The ovarian tumor cohort (*n* = 51) was also reported previously [10, 71]. The tissue samples were collected at the Department of Pathology, University Hospital, Las Palmas, Gran Canaria, Spain, from surgical operations performed between 1995 and 2005. For the purpose of the present study, only samples from serous carcinomas, the most common type of ovarian malignancy, were included. This study was conducted in accordance with the Spanish codes of conduct (Ley de Investigación Biomédica) and the World Medical Association Declaration of Helsinki, following approval by the review board of the participating institution, the University Hospital, Las Palmas, Gran Canaria, Spain. Informed consent for study participation and for the use of tissue samples for research was obtained from the participants in accordance with institutional and national ethical guidelines.

### Statistical analysis

Statistical analyses were performed using GraphPad Prism 9 (GraphPad Software), unless otherwise specified. Comparisons between two groups were performed using two-tailed paired or unpaired *t*-tests, depending on whether the biological replicates were matched or independent. Experiments involving two independent variables were analyzed using a two-way ANOVA followed by Šídák’s multiple-comparisons test. For RNA-seq data (Fig. 2e, f), the expression levels were log_2_-transformed for visualization, and significance was determined by the adjusted *P* values calculated using DESeq2. In Fig. 2i, the proportions of all cell-cycle phases (G1, S, and G2/M) were normalized to the total cell-cycle population, with each phase calculated as its percentage divided by (G1% + S% + G2/M%). For quantitative data, values are presented as mean ± SD unless otherwise specified. Where shown, individual data points represent biological replicates. Statistical significance was defined as follows: *P* < 0.05 (*), *P* < 0.01 (**), *P* < 0.001 (***), and *P* < 0.0001 (****).

### Ethics statement

The use of archival formalin-fixed, paraffin-embedded human tissue samples in this study was reviewed and approved by the Ethics Committee of the University Hospital of Las Palmas, Gran Canaria, Spain. The study was conducted in accordance with the Spanish Ley de Investigación Biomédica and the World Medical Association Declaration of Helsinki. Informed consent for study participation and for the use of tissue samples for research was obtained from the participants in accordance with institutional and national ethical guidelines. No identifiable personal data, images, or videos of individual participants are included in this manuscript. All animal experiments were reviewed and approved by the Zhejiang University Laboratory Animal Welfare and Ethics Review Committee under ethical certificate ZJU20240811, and were carried out in accordance with institutional and national guidelines for the care and use of laboratory animals.

## Supplementary information

Supplementary Table

Original Data

Supplementary Figure

Supplementary Figure Legend

## Data Availability

The sequencing data supporting the findings of this study have been deposited in the NCBI Sequence Read Archive (SRA) under BioProject ID: PRJNA1418014. Source data are provided with this paper, and full-length uncropped original western blots are provided in the file Original Data. Other data are available from the corresponding authors upon reasonable request.
