# Supplementary material for: Nucleoporin TPR integrates MAPK signaling with mitogen-induced transcriptional programs
Source: Cell Death Dis. 2026 Apr 24;17(1):400. doi: 10.1038/s41419-026-08760-8 (PMC13109368; doi:10.1038/s41419-026-08760-8)
Supplement: Supplementary file 1 — Supplementary Table [file 41419_2026_8760_MOESM1_ESM.pdf]

Nucleoporin TPR Integrates MAPK Signaling with Mitogen-Induced Transcriptional Programs

Jin Liu[1,2], Yifan Zheng[1,2+], Yi Xiong[1,2+], Runhua Ma[1,2], Zihao Lin[1,2], Haikun Lin[1,2], Miguel Andújar-Sánchez[3], Jirina Bartkova[4,5], Jian Liu[6,2,7], Marco Foiani[8], Jiri Bartek[4,5*] and Martin Kosar[1,2,7*]

[1] Department of Burns, the Second Affiliated Hospital of Zhejiang University School of Medicine, and the Centre for Infection, Immunity, and Cancer (IIC) at Zhejiang University–University of Edinburgh Institute, Zhejiang University School of Medicine, China

[2] Edinburgh Medical School: Biomedical Sciences, College of Medicine and Veterinary Medicine, The University of Edinburgh, Edinburgh, UK

[3] Pathology Department, Complejo Hospitalario Universitario Insular Materno Infantil, Las Palmas de Gran Canaria, Spain

[4] Division of Genome Biology, Department of Medical Biochemistry and Biophysics, Science for Life Laboratory, Karolinska Institutet, Stockholm, Sweden

[5] Danish Cancer Institute, Danish Cancer Society, Copenhagen, Denmark

[6] Centre for Infection, Immunity, and Cancer (IIC) at Zhejiang University–University of Edinburgh Institute, Zhejiang University School of Medicine, China

[7] Biomedical and Health Translational Research Center of Zhejiang Province, Haining, China

[8] IFOM, Fondazione Istituto FIRC di Oncologia Molecolare, Milano, Italy

Supplemental Table 1

Primers and siRNA

Supplemental Table 1

The following primers are used for qPCR		
Sequence	Name	Description
5’-ACAACTTTGGTATCGTGGAAGG-3’	GAPDH_F	Human
5’-GCCATCACGCCACAGTTTC-3’	GAPDH_R	Human
5’-CCAACCGCGAGAAGATGA-3’	ACTB_F	Human
5’-CCAGAGGCGTACAGGGATAG-3’	ACTB_R	Human
5’-AGTTGGGACCACCAGTTCAG-3’	TPR_F	Human
5’-GTCAACTGAAGGCCACGTC-3’	TPR_R	Human
5’-CCGGGGATAGCCTCTCTTACT-3’	FOS_F	Human
5’-CCAGGTCCGTGCAGAAGTC-3’	FOS_R	Human
5’-ATCGGCTACGTCATCAACGTC-3’	DUSP10_F	Human
5’-TCATCCGAGTGTGCTTCATCA-3’	DUSP10_R	Human
5’-GGAATCCAAAACCGTGGAGTAA-3’	ONECUT2_F	Human
5’-CTCTTTGCGTTTGCACGCTG-3’	ONECUT2_R	Human
5’-ATGCCTTCCCACCTTGTGAG-3’	RBM15_F	Human
5’-GGTCAGCGCCAAGTTTTCTC-3’	RBM15_R	Human
5’-GCTACCCCACCGAGTACATC-3’	RHOU_F	Human
5’-GGCTCACGACACTGAAGCA-3’	RHOU_R	Human
5'-TTCAGAACATGCCTCTCAGTCG-3'	Tpr_F	Mouse
5'-GCTGTTTCGTTTGTCGTGGATA-3'	Tpr_R	Mouse
5'-GCGAGCAACTGAGAAGAC-3'	Fos_F	Mouse
5'-TTGAAACCCGAGAACATC-3'	Fos_R	Mouse
5'-AGGTCGGTGTGAACGGATTTG-3'	Gapdh_F	Mouse
5'-TGTAGACCATGTAGTTGAGGTCA-3'	Gapdh_R	Mouse

The following primers are used for genotyping		
Sequence	Name	Description
5'-ACCTGTAGTTAGTCATAGACAGCTGG-3'	Tpr mouse genotyping primer F1	Provided by GemPharmatech
5'-CTGTTTAAGACCCTTTTATACCACTG-3'	Tpr mouse genotyping primer R1	Provided by GemPharmatech
5'-ACTTAGAGTGGGTCTGTGCAGATG-3'	Tpr mouse genotyping primer F2	Provided by GemPharmatech
5'-AGGGACCAAAGGCCATGTTG-3'	Tpr mouse genotyping primer R2	Provided by GemPharmatech

Supplemental Table 1

siRNA				
Sequence	Name	siRNA	Source	Identifier
5'-GAGUCUGCGUUAUCGACAAtt-3’	siTPR53	Silencer Select siRNA against TPR #53	Thermo Fisher Scientific	siRNA ID: s14353
5'-UUGUCGAUAACGCAGACUCtc-3’	siTPR53	Silencer Select siRNA against TPR #53	Thermo Fisher Scientific	siRNA ID: s14353
5'-GAAGUUCAUACUAAGCGUAtt-3’	siTPR54	Silencer Select siRNA against TPR #54	Thermo Fisher Scientific	siRNA ID: s14354
5'-UACGCUUAGUAUGAACUUCct-3’	siTPR54	Silencer Select siRNA against TPR #54	Thermo Fisher Scientific	siRNA ID: s14354
5'-CAAUAUGAAGGUCGAAUUAtt-3’	siTPR55	Silencer Select siRNA against TPR #55	Thermo Fisher Scientific	siRNA ID: s14355
5'-UAAUUCGACCUUCAUAUUGgg-3’	siTPR55	Silencer Select siRNA against TPR #55	Thermo Fisher Scientific	siRNA ID: s14355
5'-GAUAAUCGAAGAAUGGAAAtt-3’	siTPR1	Silencer Select siRNA against TPR #1	Tsingke	
5'-UUUCCAUUCUUCGAUUAUCtt-3’	siTPR1	Silencer Select siRNA against TPR #1	Tsingke	
5'-GGAUCAGCUCAACCACAUUtt-3'	siERK1	Silencer Select siRNA against MAPK3	Thermo Fisher Scientific	siRNA ID: s11140
5'-AAUGUGGUUGAGCUGAUCCag-3'	siERK1	Silencer Select siRNA against MAPK3	Thermo Fisher Scientific	siRNA ID: s11140
5'-CAGGGUUCCUGACAGAAUAtt-3'	siERK2	Silencer Select siRNA against MAPK1	Thermo Fisher Scientific	siRNA ID: s11137
5'-UAUUCUGUCAGGAACCCUGtg-3'	siERK2	Silencer Select siRNA against MAPK1	Thermo Fisher Scientific	siRNA ID: s11137
