# Supplementary material for: Nucleoporin TPR integrates MAPK signaling with mitogen-induced transcriptional programs
Source: Cell Death Dis. 2026 Apr 24;17(1):400. doi: 10.1038/s41419-026-08760-8 (PMC13109368; doi:10.1038/s41419-026-08760-8)

Nucleoporin TPR Integrates MAPK Signaling with Mitogen-Induced Transcriptional Programs

Jin Liu[1,2], Yifan Zheng[1,2+], Yi Xiong[1,2+], Runhua Ma[1,2], Zihao Lin[1,2], Haikun Lin[1,2], Miguel Andújar-Sánchez[3], Jirina Bartkova[4,5], Jian Liu[6,2,7], Marco Foiani[8], Jiri Bartek[4,5*] and Martin Kosar[1,2,7*]

[1] Department of Burns, the Second Affiliated Hospital of Zhejiang University School of Medicine, and the Centre for Infection, Immunity, and Cancer (IIC) at Zhejiang University–University of Edinburgh Institute, Zhejiang University School of Medicine, China

[2] Edinburgh Medical School: Biomedical Sciences, College of Medicine and Veterinary Medicine, The University of Edinburgh, Edinburgh, UK

[3] Pathology Department, Complejo Hospitalario Universitario Insular Materno Infantil, Las Palmas de Gran Canaria, Spain

[4] Division of Genome Biology, Department of Medical Biochemistry and Biophysics, Science for Life Laboratory, Karolinska Institutet, Stockholm, Sweden

[5] Danish Cancer Institute, Danish Cancer Society, Copenhagen, Denmark

[6] Centre for Infection, Immunity, and Cancer (IIC) at Zhejiang University–University of Edinburgh Institute, Zhejiang University School of Medicine, China

[7] Biomedical and Health Translational Research Center of Zhejiang Province, Haining, China

[8] IFOM, Fondazione Istituto FIRC di Oncologia Molecolare, Milano, Italy

Uncropped Original Western Blots

Figure 3b

b

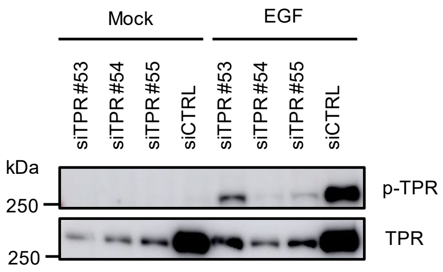
Mock
EGF
siTPR #53
siTPR #54
siTPR #55
siCTRL
siTPR #53
siTPR #54
siTPR #55
siCTRL
kDa
250
p-TPR
250
TPR

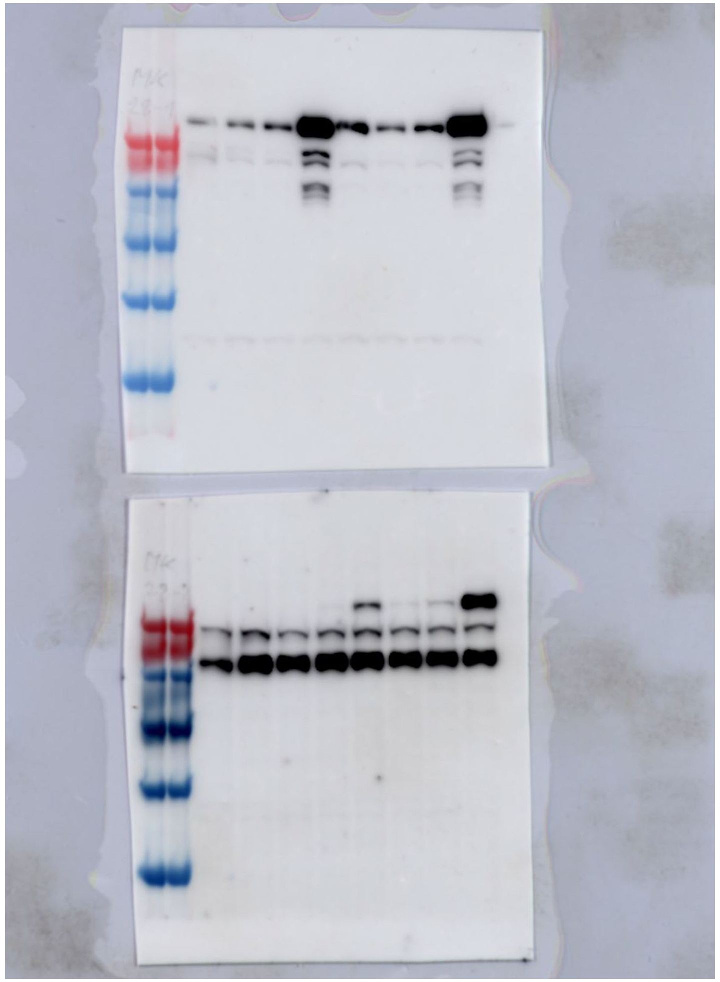

Figure 3e

e

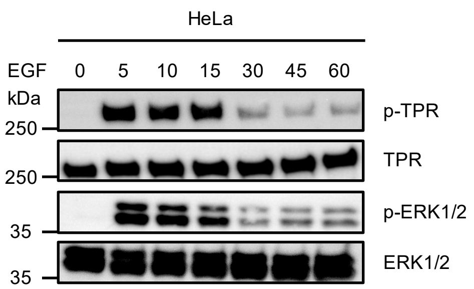
HeLa
EGF
0
5
10
15
30
45
60
kDa
250
250
35
35
p-TPR
TPR
p-ERK1/2
ERK1/2

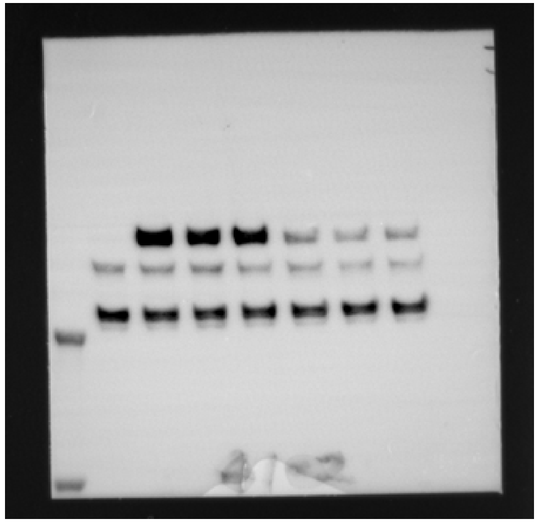

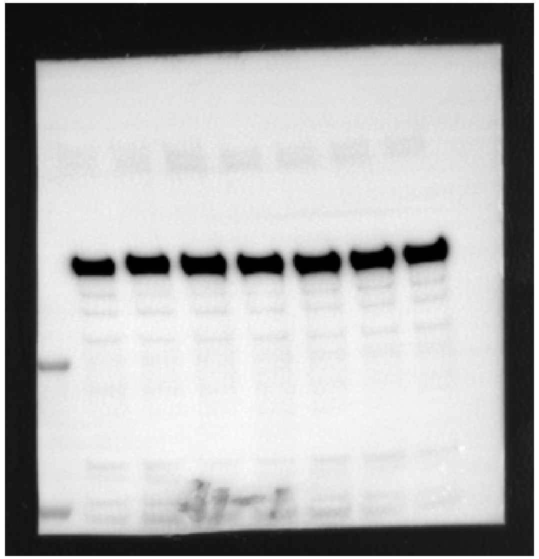

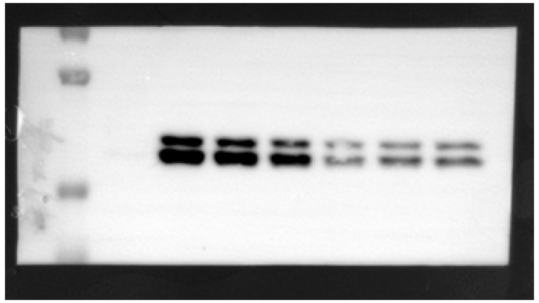

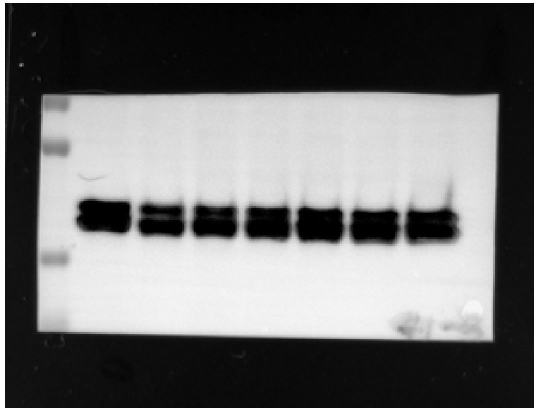

Figure 3f

f

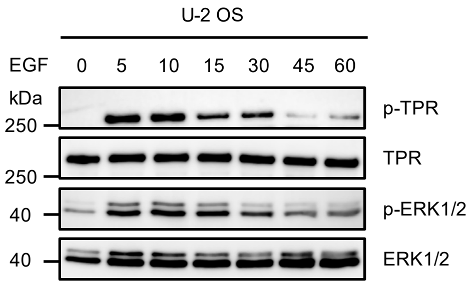
U-2 OS
EGF 0 5 10 15 30 45 60
kDa
250
250
40
40
p-TPR
TPR
p-ERK1/2
ERK1/2

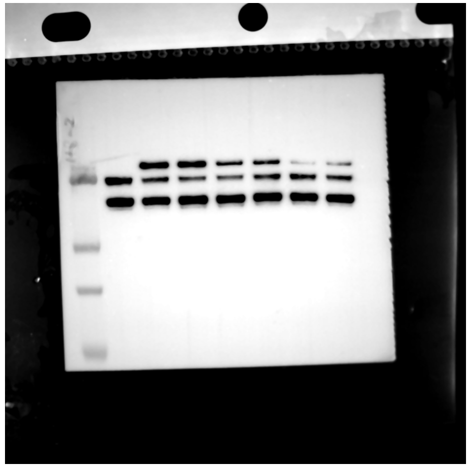

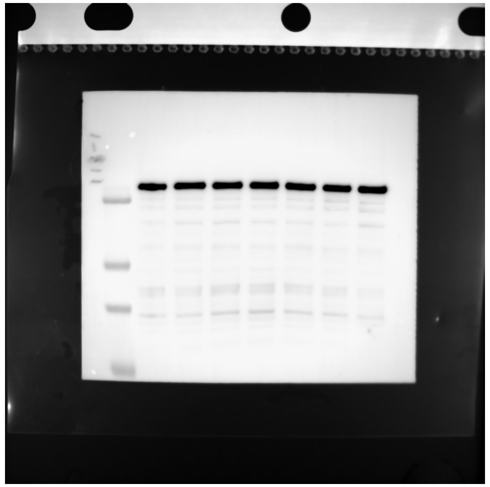

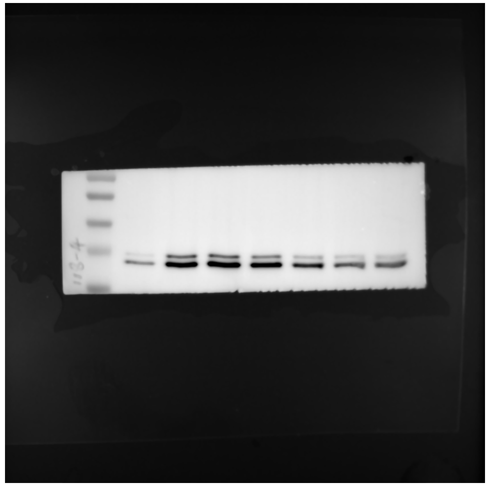

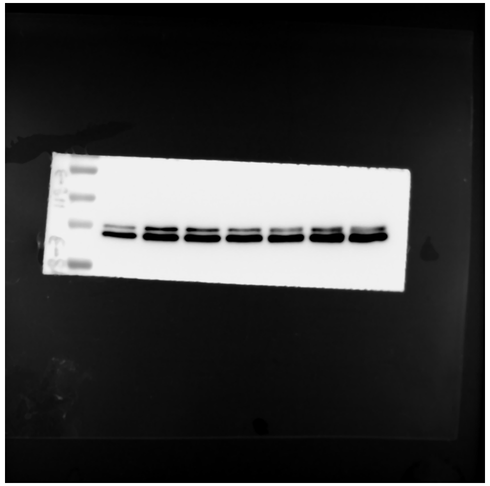

Figure 3g

g

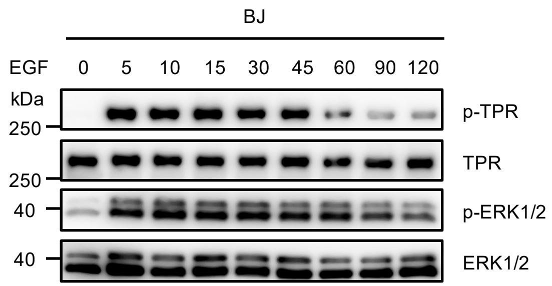
BJ
EGF 0 5 10 15 30 45 60 90 120
kDa
250
250
40
40
p-TPR
TPR
p-ERK1/2
ERK1/2

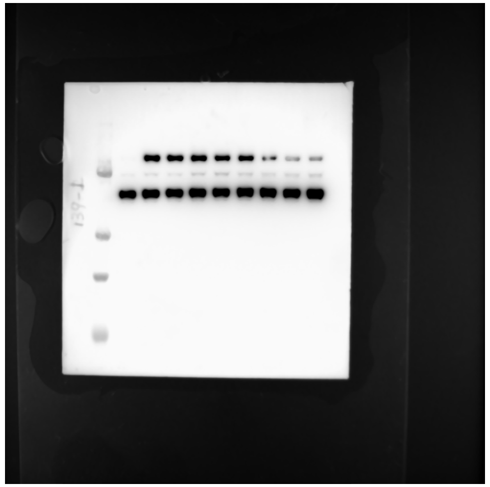

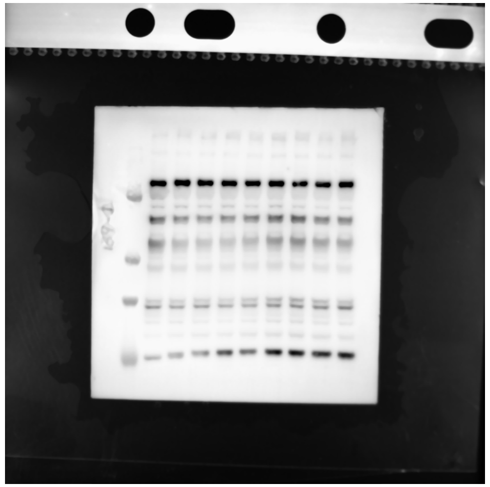

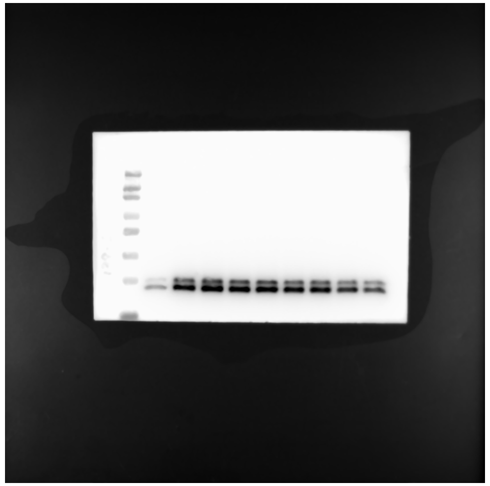

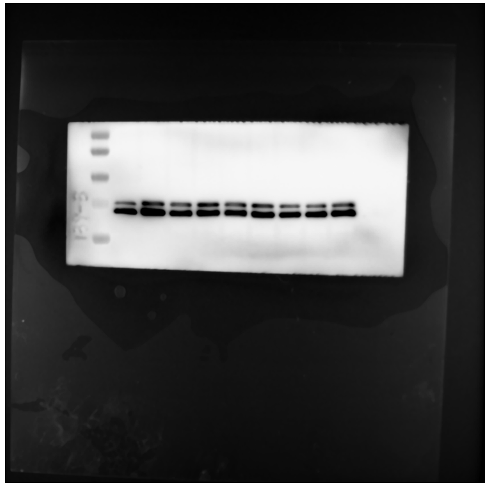

Supplementary Figure 1b

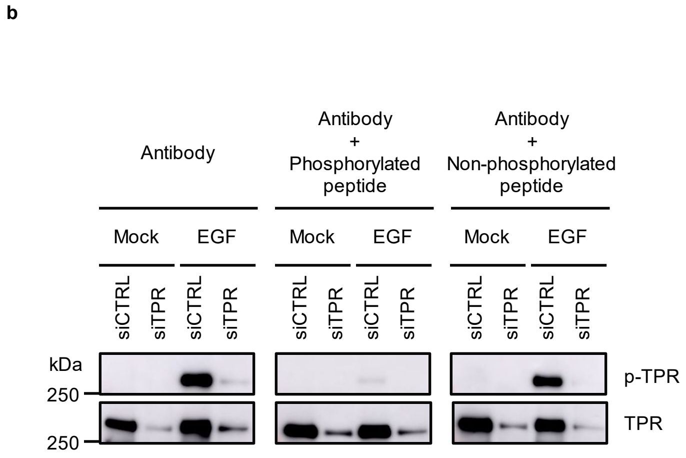

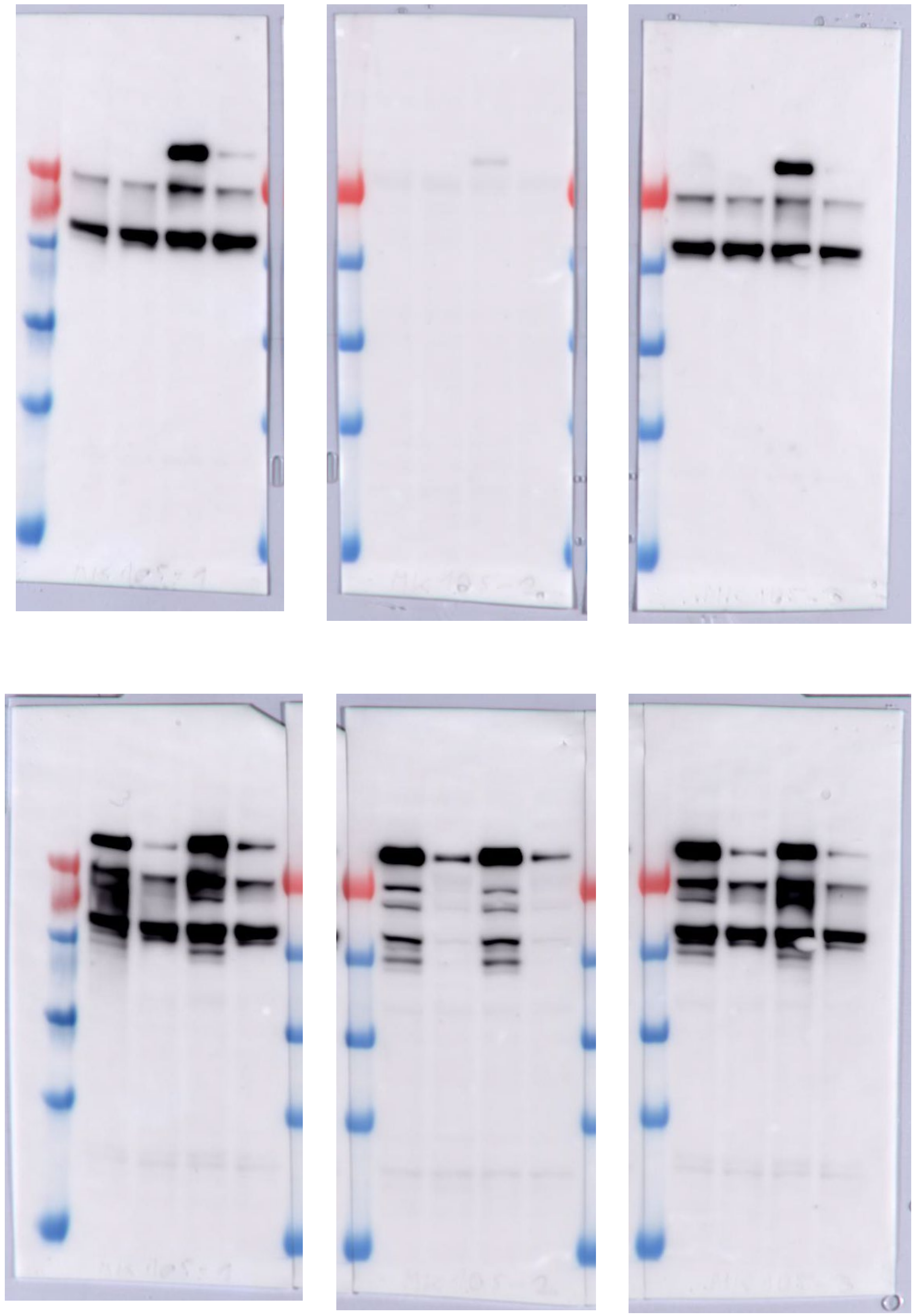

Figure 4a

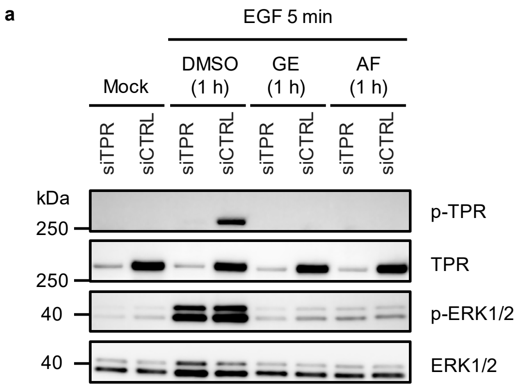
a
EGF 5 min
Mock
DMSO (1 h)
GE (1 h)
AF (1 h)
siTPR
siCTRL
siTPR
siCTRL
siTPR
siCTRL
siTPR
siCTRL
kDa
250
250
40
40
p-TPR
TPR
p-ERK1/2
ERK1/2

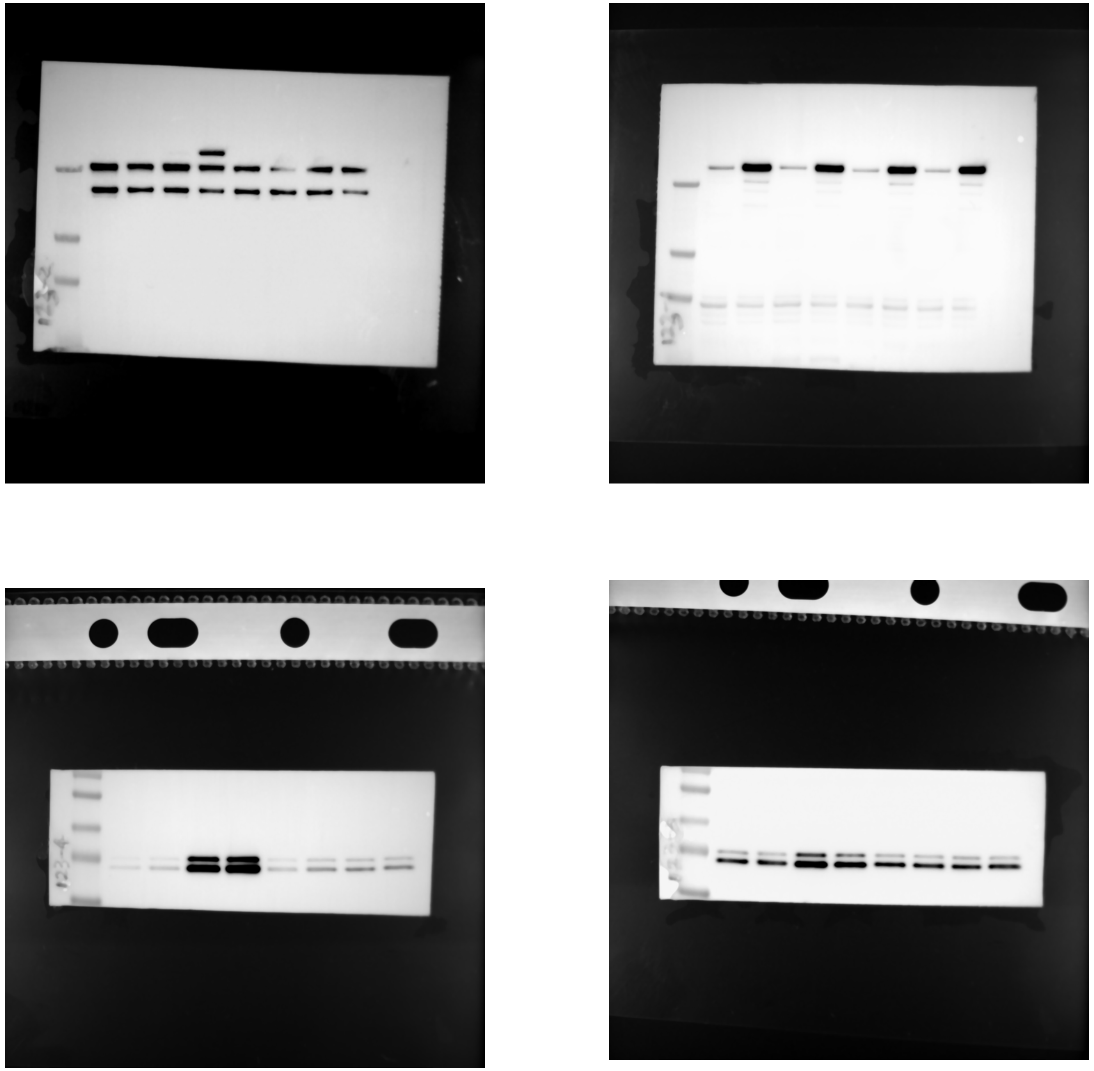

Figure 4b

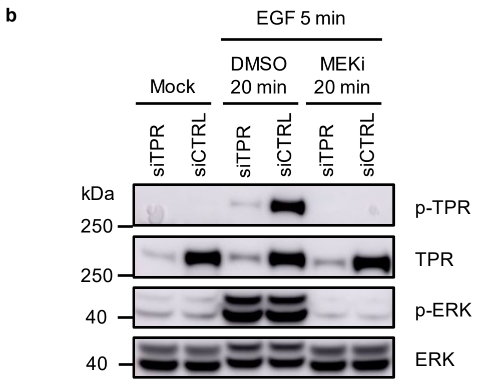
b
EGF 5 min
Mock
DMSO 20 min
MEKi 20 min
siTPR
siCTRL
siTPR
siCTRL
siTPR
siCTRL
kDa
250
250
40
40
p-TPR
TPR
p-ERK
ERK

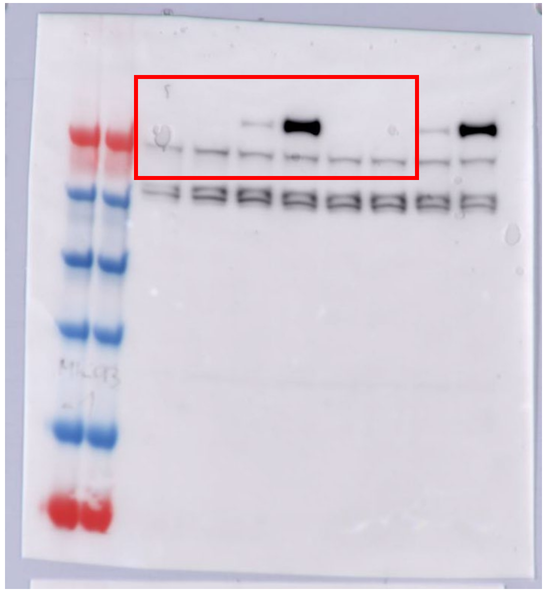

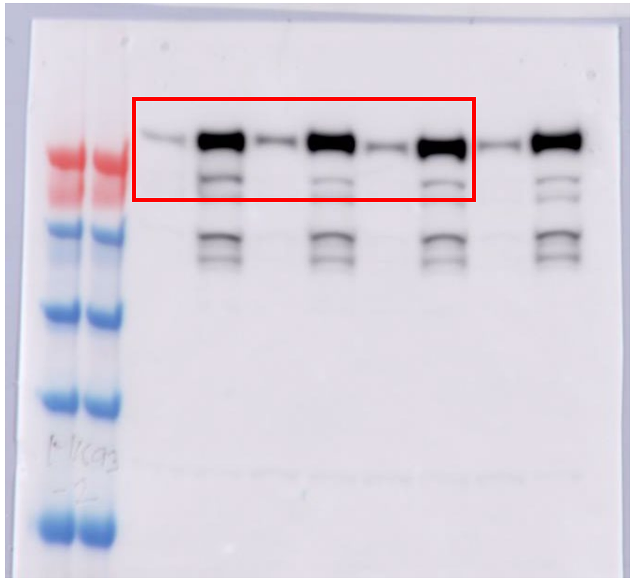

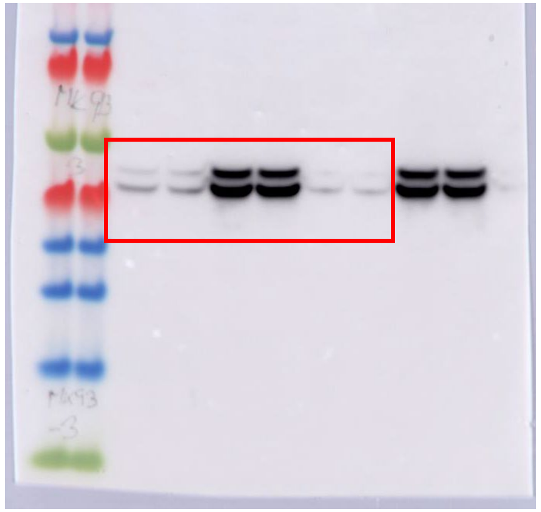

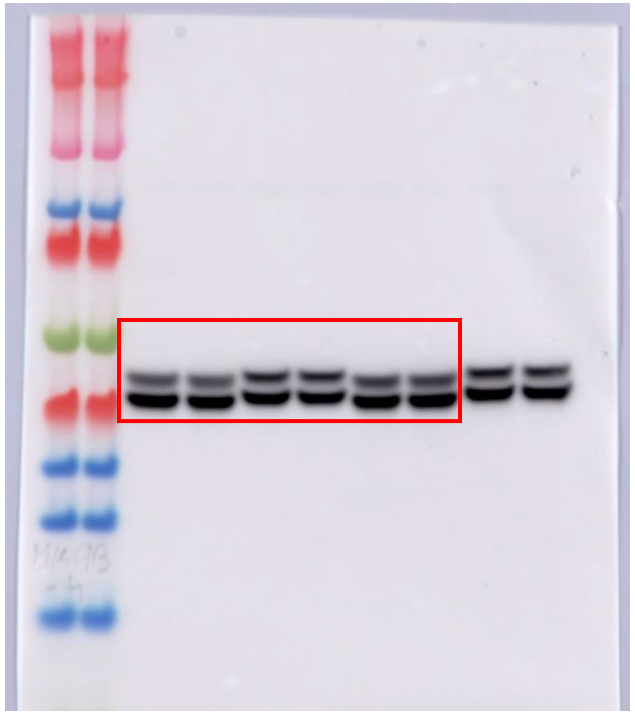

Figure 4c

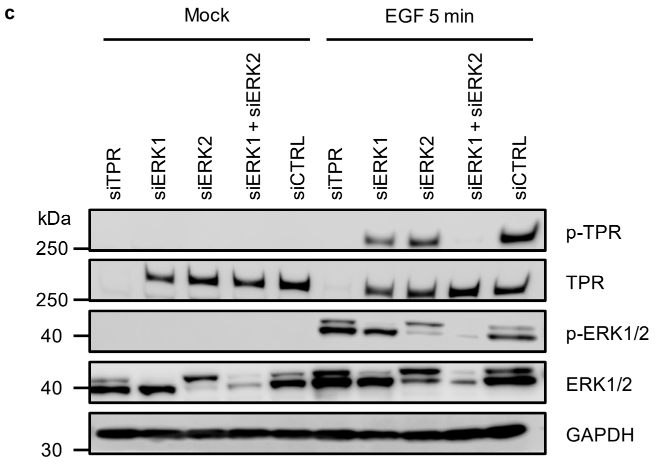
c
Mock
EGF 5 min
siTPR
siERK1
siERK2
siERK1 + siERK2
siCTRL
siTPR
siERK1
siERK2
siERK1 + siERK2
siCTRL
kDa
250
250
40
40
30
p-TPR
TPR
p-ERK1/2
ERK1/2
GAPDH

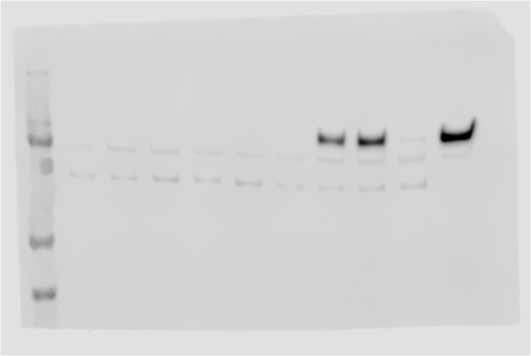

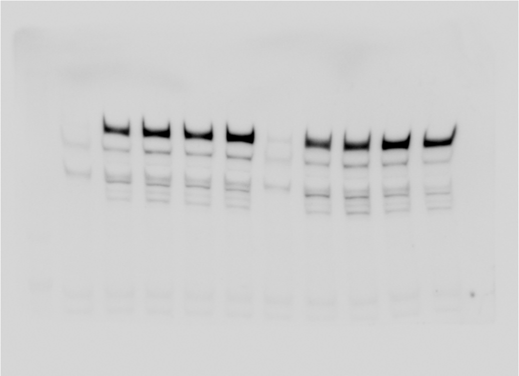

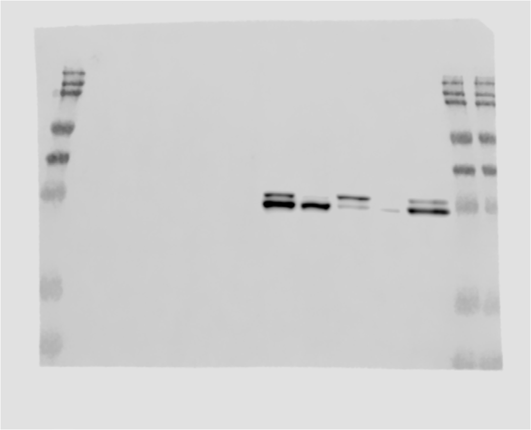

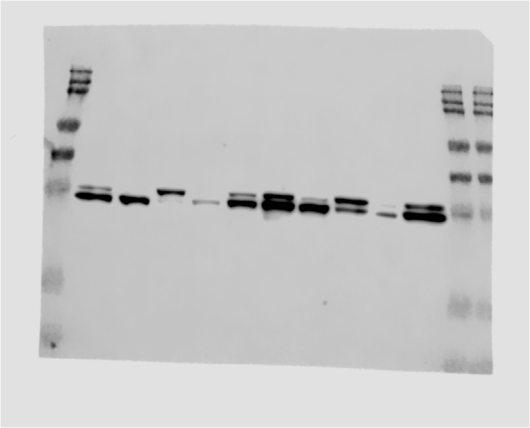

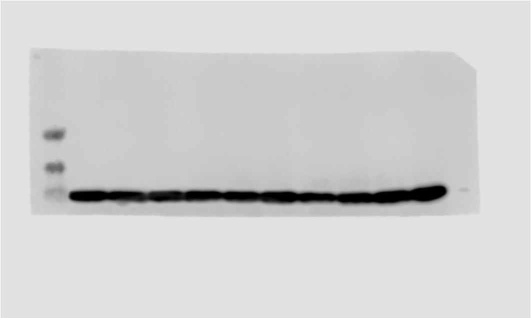

Figure 4d

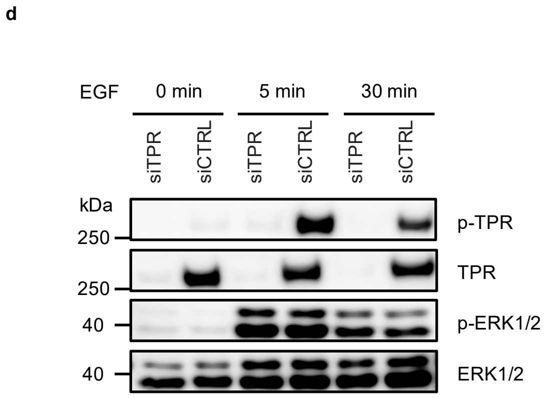
d
EGF
0 min
5 min
30 min
siTPR
siCTRL
siTPR
siCTRL
siTPR
siCTRL
kDa
250
250
40
40
p-TPR
TPR
p-ERK1/2
ERK1/2

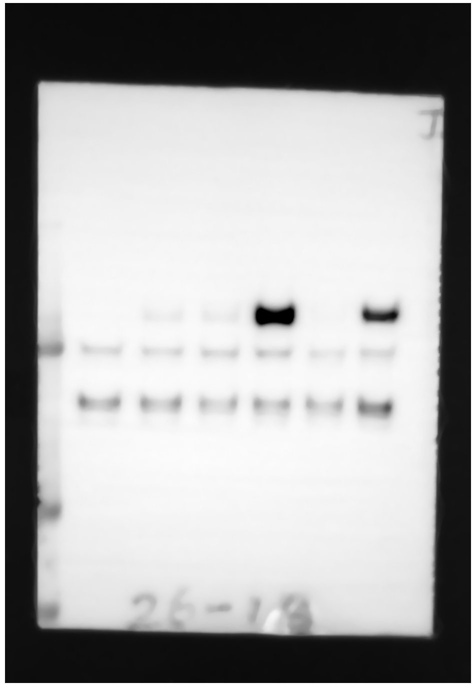

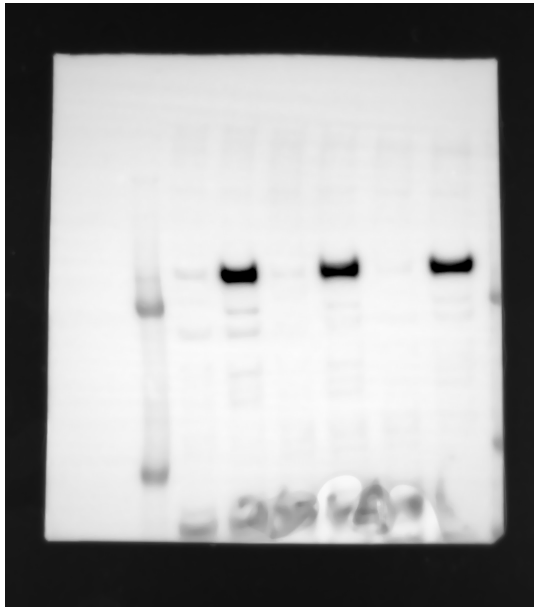

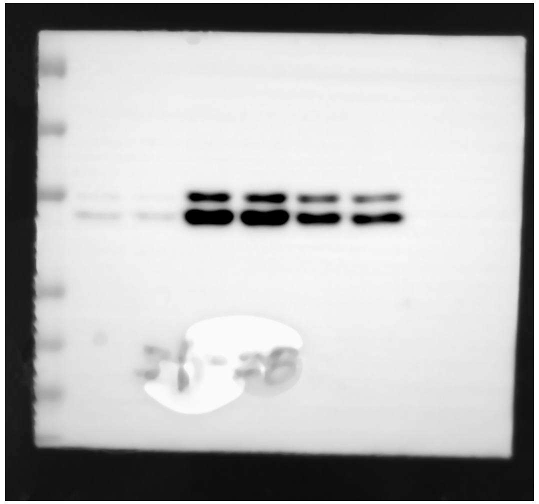

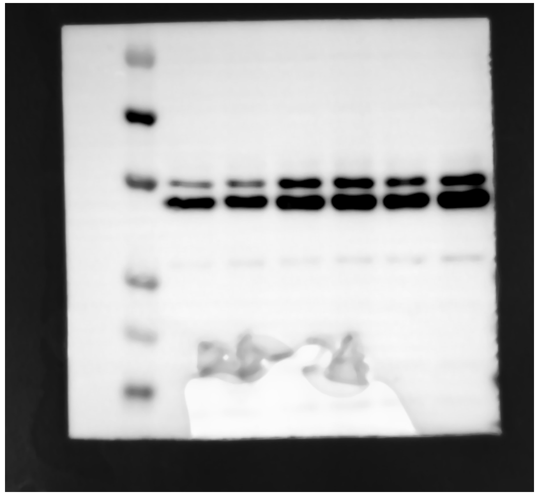

Figure 4e

e

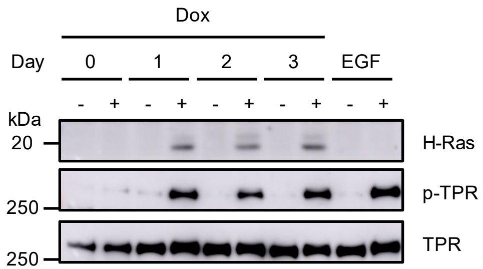

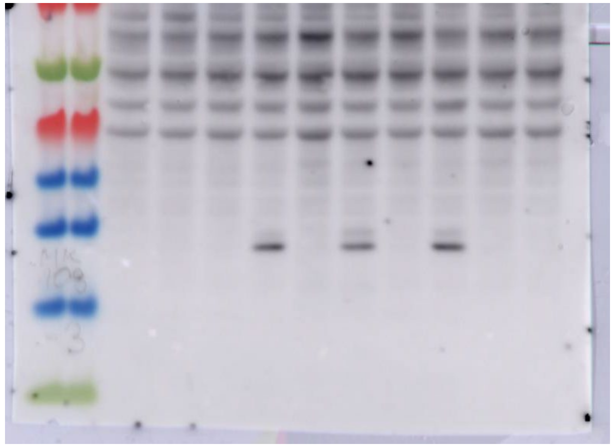

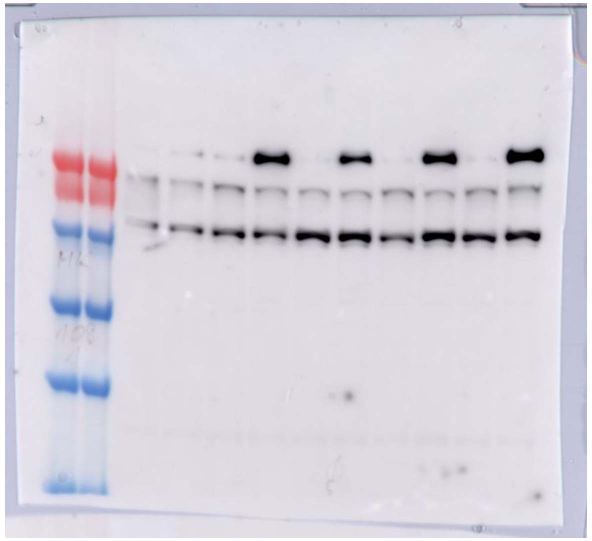

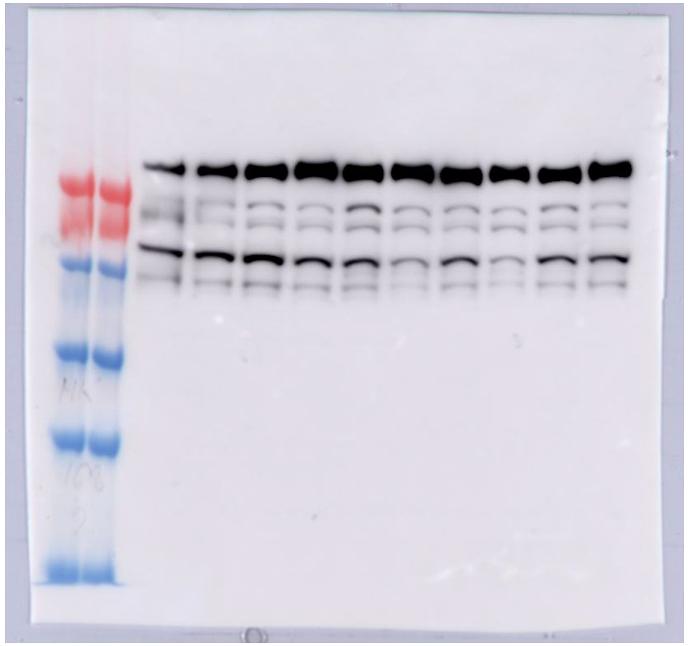

Figure 4f

f

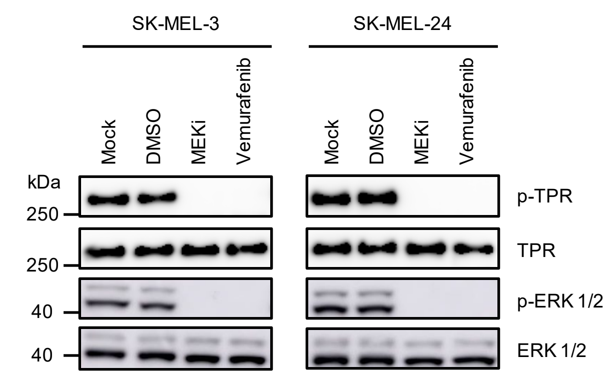
SK-MEL-3
SK-MEL-24
Mock
DMSO
MEKi
Vemurafenib
Mock
DMSO
MEKi
Vemurafenib
kDa
250
250
40
40
p-TPR
TPR
p-ERK 1/2
ERK 1/2

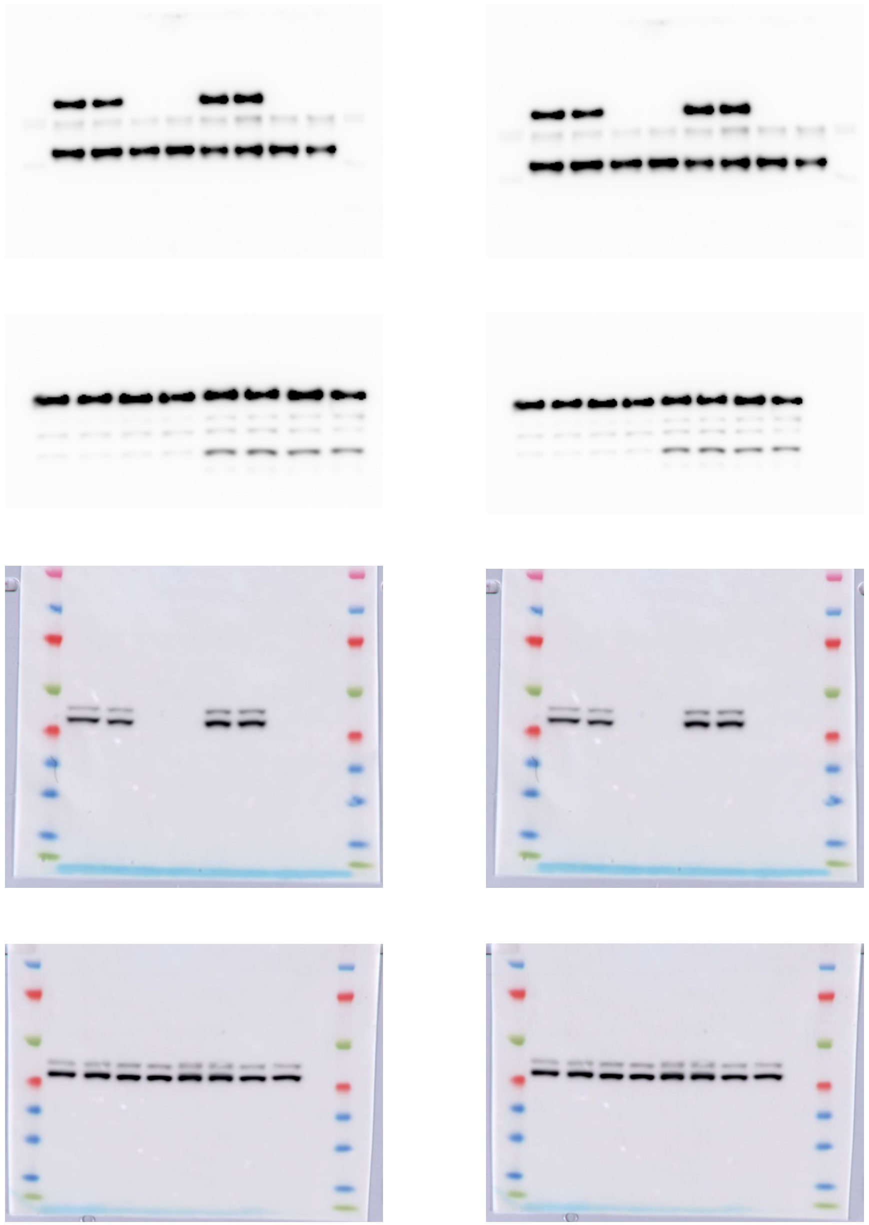

Figure 5e

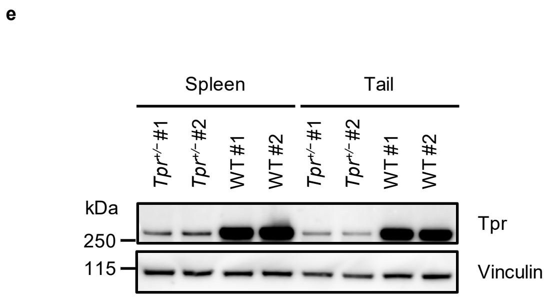
e
Spleen
Tail
Tpr+/- #1
Tpr+/- #2
WT #1
WT #2
Tpr+/- #1
Tpr+/- #2
WT #1
WT #2
kDa
250
115
Tpr
Vinculin

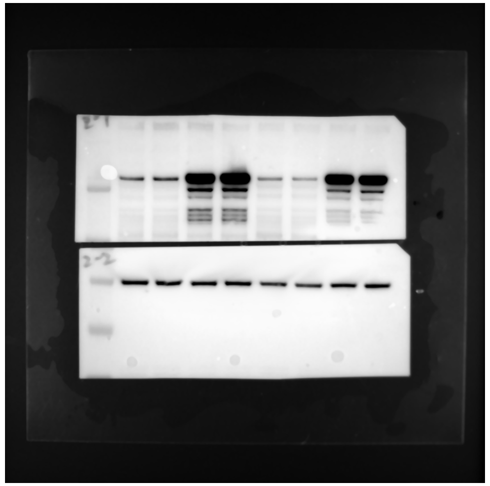

Supplement: Supplementary file 2 — Original Data [file 41419_2026_8760_MOESM2_ESM.pdf]
