# Supplementary figures and images for: Nucleoporin TPR integrates MAPK signaling with mitogen-induced transcriptional programs
Source: Cell Death Dis. 2026 Apr 24;17(1):400. doi: 10.1038/s41419-026-08760-8 (PMC13109368; doi:10.1038/s41419-026-08760-8)

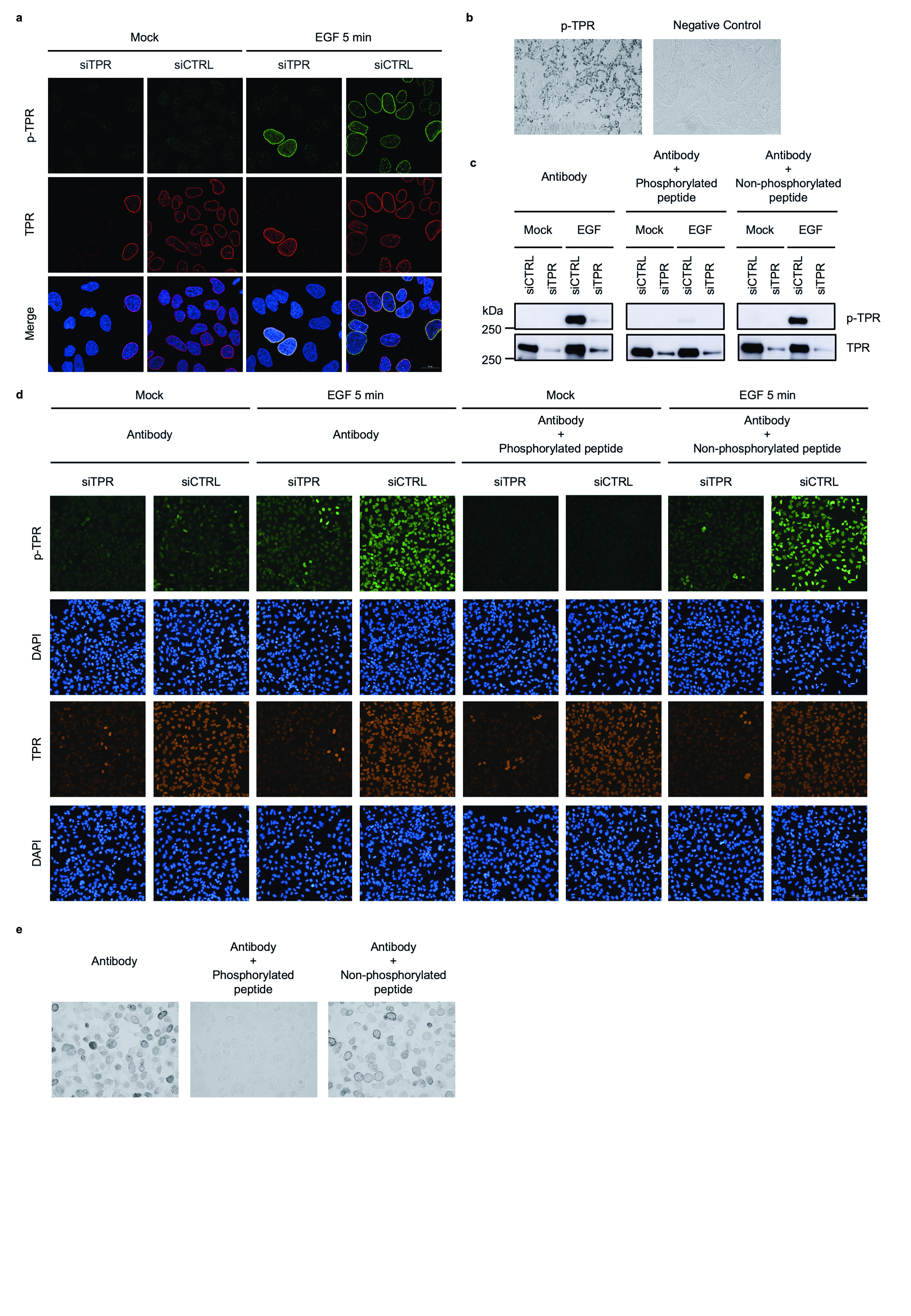

Supplement: Supplementary file 3 — Supplementary Figure [file 41419_2026_8760_MOESM3_ESM.tif]
