# Supplementary material for: Nucleoporin TPR integrates MAPK signaling with mitogen-induced transcriptional programs
Source: Cell Death Dis. 2026 Apr 24;17(1):400. doi: 10.1038/s41419-026-08760-8 (PMC13109368; doi:10.1038/s41419-026-08760-8)
Supplement: Supplementary file 4 — Supplementary Figure Legend [file 41419_2026_8760_MOESM4_ESM.docx]

**Supplementary Fig. 1: Validation of TPR phosphorylation specificity in response to EGF stimulation.**

**(a)** Confocal immunofluorescence images of HeLa cells transfected with TPR siRNA (siTPR) or control siRNA (siCTRL) under mock conditions or following EGF stimulation for 5 min. Cells were stained with antibodies against phospho-TPR-Ser2155 (p-TPR, green) and total TPR (red); nuclei were counterstained with DAPI (blue). Merged images are shown in the bottom row. Images are representative of three independent experiments. Scale bar: 10 µm.

**(b)** Human breast cancer sections were stained with the murine phospho-TPR-Ser2155 (p-TPR) antibody (left) or with normal murine serum as a negative-control primary reagent (right), followed by optimized immunoperoxidase detection. Scale bar: 50 µm.

**(c)** Immunoblot analysis of phospho-TPR-Ser2155 (p-TPR) and total TPR in HeLa cells transfected with control siRNA (siCTRL) or TPR siRNA (siTPR) and treated with mock or EGF for 5 min. The specificity of the p-TPR signal was confirmed by pre-incubation of the p-TPR antibody with either a phosphorylated or a non-phosphorylated peptide corresponding to the TPR phosphorylation site. Total TPR served as a loading control. Data are representative of three independent experiments.

**(d)** Immunofluorescence microscopy showing the nuclear localization of phospho-TPR-Ser2155 (p-TPR, green) and total TPR (orange) in siTPR- and siCTRL-transfected HeLa cells treated with 5 min EGF stimulation or mock control. Antibody specificity was tested by pre-incubating the phospho-TPR (Ser2155) antibody with either a phosphorylated or a non-phosphorylated peptide prior to staining. DAPI (blue) was used to visualize nuclei. Scale bar: 100 µm.

**(e)** Immunohistochemical staining of phospho-TPR-Ser2155 (p-TPR) in paraffin-embedded MDA-MB-231 breast cancer cells. The phospho-TPR-Ser2155 antibody was pre-incubated with either a phosphorylated or a non-phosphorylated peptide to confirm antibody specificity. Scale bar: 10 µm.
